# Asymmetric cellular memory in bacteria exposed to antibiotics

**DOI:** 10.1186/s12862-017-0884-4

**Published:** 2017-03-09

**Authors:** Roland Mathis, Martin Ackermann

**Affiliations:** 1 Department of Environmental Systems Science, ETH Zurich (Swiss Federal Institute of Technology in Zurich), Universitaetsstrasse 16, 8092 Zurich, Switzerland; 2 Eawag (Swiss Federal Institute of Aquatic Science and Technology), Ueberlandstrasse 133, 8600, Duebendorf, Switzerland

**Keywords:** Bacterial memory, Priming, Caulobacter crescentus, Asymmetry

## Abstract

**Background:**

The ability to form a cellular memory and use it for cellular decision-making could help bacteria to cope with recurrent stress conditions. We analyzed whether bacteria would form a cellular memory specifically if past events are predictive of future conditions. We worked with the asymmetrically dividing bacterium *Caulobacter crescentus* where past events are expected to only be informative for one of the two cells emerging from division, the sessile cell that remains in the same microenvironment and does not migrate.

**Results:**

Time-resolved analysis of individual cells revealed that past exposure to low levels of antibiotics increases tolerance to future exposure for the sessile but not for the motile cell. Using computer simulations, we found that such an asymmetry in cellular memory could be an evolutionary response to situations where the two cells emerging from division will experience different future conditions.

**Conclusions:**

Our results raise the question whether bacteria can evolve the ability to form and use cellular memory *conditionally* in situations where it is beneficial.

**Electronic supplementary material:**

The online version of this article (doi:10.1186/s12862-017-0884-4) contains supplementary material, which is available to authorized users.

## Background

In bacteria, as in every cellular organism, the current state and composition of a cell is determined by events in the past [1–4]. Factors like nutrient availability, physical and chemical conditions and biological signals in the recent past influence the current set of transcripts and proteins in a cell, its metabolic activity, and other aspects of its phenotype. We refer to this dependency of the current state of single cells on past conditions as ‘cellular memory’. We use the term ‘cellular memory’ in a phenomenological sense for situations where a phenotypic trait of a cell depends on past conditions, irrespective of the underlying molecular mechanism. One fundamental question is whether cellular memory is beneficial [1, 3, 4]: To what degree does it allow a cell to better cope with current and future conditions? Do bacteria ‘remember’ past environmental states and use this memory to anticipate future conditions?

This question is motivated by the fact that the degree of cellular memory is shaped by biological processes that are encoded by genes, and that can thus undergo evolutionary change in response to natural selection. Cellular memory depends on the half-lives and turnover of cellular components—for example RNAs and proteins—and on the architecture of gene-regulatory networks [1, 5–7]. Importantly, cellular memory can be differentially controlled for different phenotypic traits: for example, traits that are based on transcripts with long half-lives and stable proteins are expected to show a large degree of cellular memory, whereas other traits that are based on transcripts with short half-lives and unstable proteins are expected to have a low degree of cellular memory.

The ‘adaptive value’ of cellular memory—its effect on survival and reproduction of individual cells—is expected to depend on the specific situation. For some environmental factors the conditions in the past might be indicative of the conditions in the future, and cellular memory for phenotypic traits that determine the response to these environmental factors might thus be beneficial; for other factors this might not be the case. This raises the question whether bacteria (and other organisms) evolved high degrees of cellular memory specifically in traits and in circumstances where this is beneficial, and low degrees of cellular memory when it is not. We refer to this possibility as *conditional* cellular memory.

We investigated if organisms memorize past events specifically in situations where past events have predictive value about the future. To address this question, we used an experimental system where cellular memory is expected to be beneficial to some cells in a population, and less beneficial to other cells. Specifically, we worked with an asymmetrically dividing bacterium—*Caulobacter crescentus*—where one cell emerging from division remains in the same microenvironment, while the other migrates to a different microenvironment [8]. For the first cell the future conditions are expected to be related to the past (since the cell is staying in the same microenvironment), and for this cell it might therefore be advantageous to memorize past events to prepare for future conditions. For the second cell future conditions are not related, or related to a lesser degree, to the past. It could thus be neutral or disadvantageous to prepare for these future conditions based on past events. Based on these considerations, one would expect the second cell to show a lower degree of cellular memory than the first cell. If in such a scenario we can observe differences in how these two cell types use past events to prepare for future environmental conditions, this would be consistent with the interpretation that this organism has evolved the ability to memorize past events, but to use this ability only if it is beneficial (we use the phrasing ‘consistent with the interpretation’ to emphasize that such a finding would not firmly establish that asymmetric memory is a direct consequence of natural selection on this trait, since asymmetric memory could also be caused by a range of other factors such as differences in the cellular composition due to the asymmetry of mother and daughter cell in this bacterium [9]).

We chose *C. crescentus* to address this question of conditional cellular memory because it is a well-established model organism to study asymmetric cell division. A surface attached cell divides asymmetrically into a sessile stalked cell and motile swarmer cell. We refer to the sessile stalked cell as ‘mother’ and the motile swarmer cell as ‘daughter’ as for example in [10, 11]. This organism thus provides an ideal opportunity to address the question whether this organism uses cellular memory depending on whether memory helps cells to anticipate future conditions or not. While the sessile mother cell remains attached to the surface at the same location as before division, the motile daughter cell can move to a different microenvironment before differentiating into a stalked cell and initiating division. For the sessile mother information about the recent past may thus be of value to predict upcoming environmental conditions. For the motile daughter the past is arguably less informative, due to the change of location [12].

Bacterial memory has been studied in different contexts such as changing nutrient conditions [1, 13–16] or cues that indicate upcoming adverse conditions [3, 17–20]. Here we exposed cells with a low level of a stressor as a ‘warning’ of an upcoming exposure to a higher level of the same stressor. In such a scenario a cell that uses the warning to prepare for an upcoming stressful event can have a higher probability to survive. We can thus ask whether cells keep a cellular memory of the warning only if they are staying in the same microenvironment, and do not keep a cellular memory if they are migrating to another microenvironment where the timing of stress is probably different.

We used a combination of single-cell experiments and computer simulations to address these questions. We experimentally exposed *C. crescentus* to the antibiotic ampicillin [21]. *C. crescentus* carries six genes that confer—when expressed—a certain degree of resistance to ampicillin, a beta-lactam antibiotic [22]. Resistance to antibiotics has been found to be common in bacteria living in freshwater, and might be an adaptation to antibiotics produced by other microorganisms [23–27]. *C. crescentus* is potentially exposed to naturally occurring beta-lactams in its freshwater environment [28]. Antibiotics are an ideal stressor to test our hypothesis about asymmetric memory in bacteria: while a sessile bacterial cell is bound to ‘endure’ an exposure to antibiotics produced by other microbes in the same microenvironment; a motile cell, in contrast, might move away from these producers and into a new microenvironment with a different regime of antibiotics exposure. We thus analyzed, for both cell types, whether a previous warning event would increase the tolerance to a subsequent stress event. These experiments indeed revealed a small but statistically significant asymmetry in the distribution of the cellular memory in response to antibiotics: the warning increased the survival of the sessile mother but not of the motile daughter during subsequent exposure to high concentrations of antibiotics.

These experimental results motivated us to ask whether such asymmetry in how past events influence future behavior could indeed be an evolved response to a situation where the predictive value of past events differs for the two cell types emerging from division. We used computer simulations to test whether asymmetric memory is expected to evolve in a situation where one individual emerging from division remains in the same environment while the other individual migrates to a different environment. These simulations support the notion that the asymmetry in cellular memory that we observed in the single-cell experiments could be the result of differential selection on the sessile and motile cell type. More generally, our experimental and theoretical results suggest that analyzing what types of past events are stored in different cell types allows formulating hypotheses about the adaptive nature of bacterial memory.

## Results and discussion

### Single-cell experiments

**Fig. 1:**
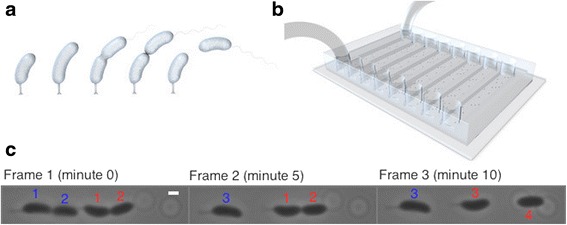
Microfluidics setup. **a** *Caulobacter crescentus* divides asymmetrically into a surface attached stalked cell (which we call mother) and a motile swarmer cell (which we call daughter). The figure shows five stages of a cell division cycle of a stalked cell (the duration of the cell cycle of a stalked cell is about 60 min). **b** Experiments were conducted using microfluidic chips with eight parallel channels. Environmental conditions were controlled by flowing medium through the channels (tubing shown for one channel). Stalked cells attached to the glass surface at the inside of the chip. Upon division, and for the *C. crescentus* strain used in this study, about one third of *C. crescentus* swarmer cells attached downstream of the stalked cell and were observable, while the other swarmer cells were washed out. **c** *C. crescentus* cells were monitored with time-lapse microscopy during growth under controlled conditions. Images were acquired every 5 min. The stalked mother cells divided asymmetrically about once an hour into stalked mother and motile daughter cells. Three successive images (minute 0, 5, 10) of two cell lineages (*blue* and *red*) are shown. Flow direction is from left to right. The blue cell divided between Frame 1 and Frame 2, the red cell divided between Frame 2 and Frame 3. The pre-divisional mother cells (blue cell: Frame 1. red cell: Frame 1–2) consists of the stalked mother cell (labeled 1) and swarmer daughter cell (labeled 2). Once cell division has completed, the stalked mother cell was marked 3 and the motile daughter cell was marked 4. When the blue cell divided, the swarmer daughter cell was washed out and was not observable anymore. The swarmer daughter cell that attached and remained in the channel differentiated into a stalked cell and initiated cell division (labeled 4). Figure **a** and **b** were reproduced from [17], and produced by Stefanie Stutz (http://stephaniestutz.ch/)

To impose temporal regimes of antibiotics, we grew *C. crescentus* in microfluidic devices (Fig. 1) that allowed controlling and changing the external conditions [17, 29]. The stalked mother cells attach to the glass surface at the inside of the chip, and can be observed over a large number of consecutive divisions. About two thirds of the swarmer daughter cells are removed from the chip upon cell division, while about one third remains in the chip where they differentiate to stalked cells and can be observed over subsequent cell divisions. This set-up thus allowed testing for differences in cellular memory between stalked mother cells and the swarmer progeny they produced (after these swarmer progeny attached to the surface and differentiated into stalked cells). Our analysis and interpretation of these experiments is based on the assumption that the degree of cellular memory observed in daughter cells does not depend on whether these cells remained in the chip or were removed. In other words, we assumed that the daughter cells we could observe were representative for all daughter cells that were produced with respect to cellular memory.

**Fig. 2:**
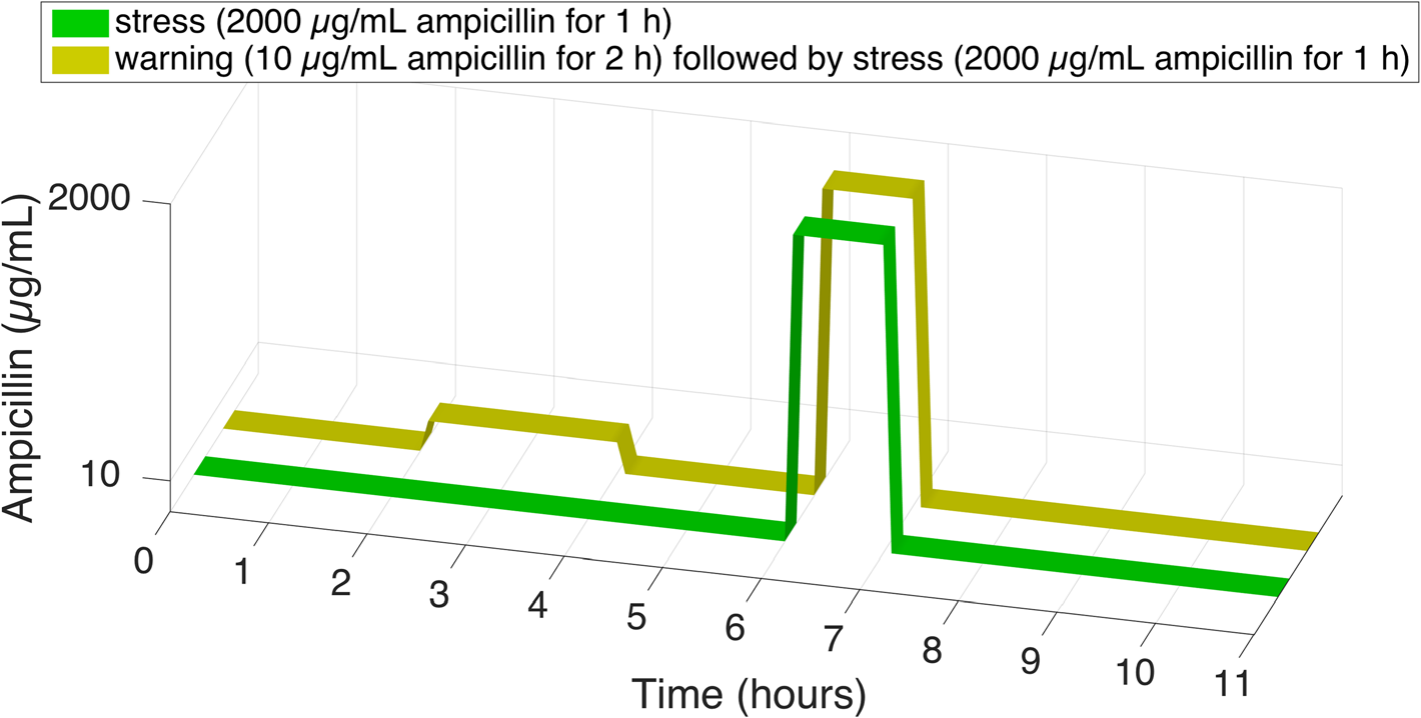
Schematic representation of the single-cell experiments. Cells were grown in a microfluidic chip for 2 h. In the ‘warning’ treatment (*yellow trajectory*) cells were then exposed to a low concentration of ampicillin (0 or 10 μg/mL) for 2 h; in the ‘no warning’ treatment (*green trajectory*) cells were not exposed to ampicillin during these 2 h. Next, cells were switched back to favorable conditions, i.e., medium without ampicillin, for 2 h. All cells were then stressed with 2000 μg/mL ampicillin for 1 h. Observation of the cells continued for 4 h after the stress event (total experiment time was 11 h)

We used the bacterium *C. crescentus* to test whether past events can prepare cells for an upcoming stress event. We exposed cells to two subsequent ampicillin events (Fig. 2, *yellow trajectory*). By exposure to a sub-MIC (minimal inhibitory concentration, 100 μg/mL in PYE broth [22]) of ampicillin we expected to activate the naturally occurring resistance mechanism of *C. crescentus* based on the expression of genes encoding beta-lactamases [22]. We refer to this as a ‘warning event’. After switching back to favorable growth conditions for a short period of time, a ‘stress event’ with a high concentration of ampicillin was imposed on the cells. In control treatments, we exposed cells to stress events without preceding warning event (Fig. 2, *green trajectory*).

**Fig. 3:**
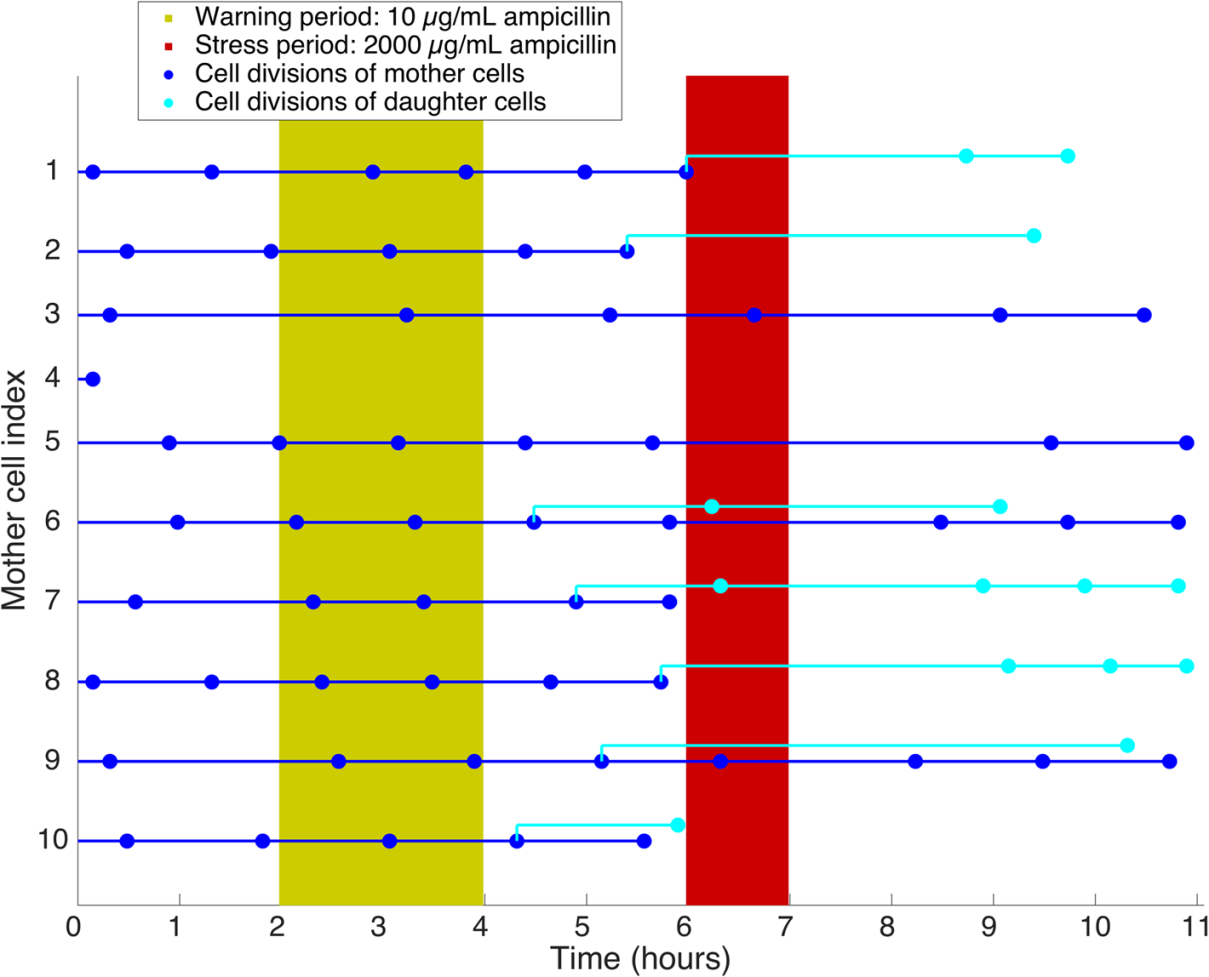
Representative single cell division trajectories. The plot shows cell divisions (*dots*) of attached *C. crescentus* cells in the microfluidic device over 11 h (2 h favorable conditions, 2 h warning conditions with 10 μg/mL ampicillin, 2 h favorable conditions, 1 h stress with 2000 μg/ml ampicillin, 4 h favorable conditions). As an example the divisions of 10 stalked mother cells (*dark blue* lines starting from the left indexed 1–10) were followed. When one of these 10 cells divided (*dark blue dot*), a stalked mother cell and a swarmer daughter cell emerged (see also Fig. 1). A continued dark blue horizontal line represents the stalked mother cell. The swarmer daughter cell either attached directly downstream of the stalked mother cell or was washed out and was no longer observable. If the swarmer daughter cell attached, a light blue line branches off from the dark blue dot representing the division event. The dots on the light blue lines represent divisions of these swarmer cells once they had differentiated into stalked cells. Horizontal lines are only shown up to the last cell division that we observed for the respective cell. We restricted the analysis of divisions to daughter cells originating from the dark blue dots in the time period between warning and stress event

We recorded and analyzed time-lapse movies of the initial cohort of sessile stalked cells and the subset of their daughters that remained in the microfluidic chip after cell division, differentiated to stalked cells and could be observed (Fig. 3, Additional file 1: Fig. S1 and Additional file 2: Fig. S2). We quantified stress tolerance by measuring the number of survivors observed after the stress event. A cell was marked as a survivor if at least one cell division was observed after exposure to the stress event. As in previous experiments with *C. crescentus* exposed to sodium chloride [17], we found that survival of the stress event depended on the cell cycle position (see Additional file 3: Figure S3). The results reported below were corrected for this effect.

We used this system to test the main idea put forward above: that the sessile mothers—but not the motile daughters—would retain a cellular memory of past events. To do so, we compared how survival under antibiotic exposure of the two groups of cells depended on a past warning. The first group (which we call ‘mothers’) was the cohort of stalked cells that were already present at the time of the warning; the second group (which we call ‘daughters’) was the cohort of cells that were produced after the warning, and that stayed in the microfluidic device and could thus be observed. It is important to note that many of the daughters had already differentiated to stalked cells at the onset of the ‘stress event’. While the time-point of differentiation for individual cells cannot be determined in our experimental set-up, the swarmer cells that remain in the microfluidic device, and that can thus be observed, only take about 20 min longer to reach the next cell division than the stalked cells (Additional file 3: Figure S3). This indicates that they have completed differentiation to a stalked cell after about 20 min.

**Fig. 4:**
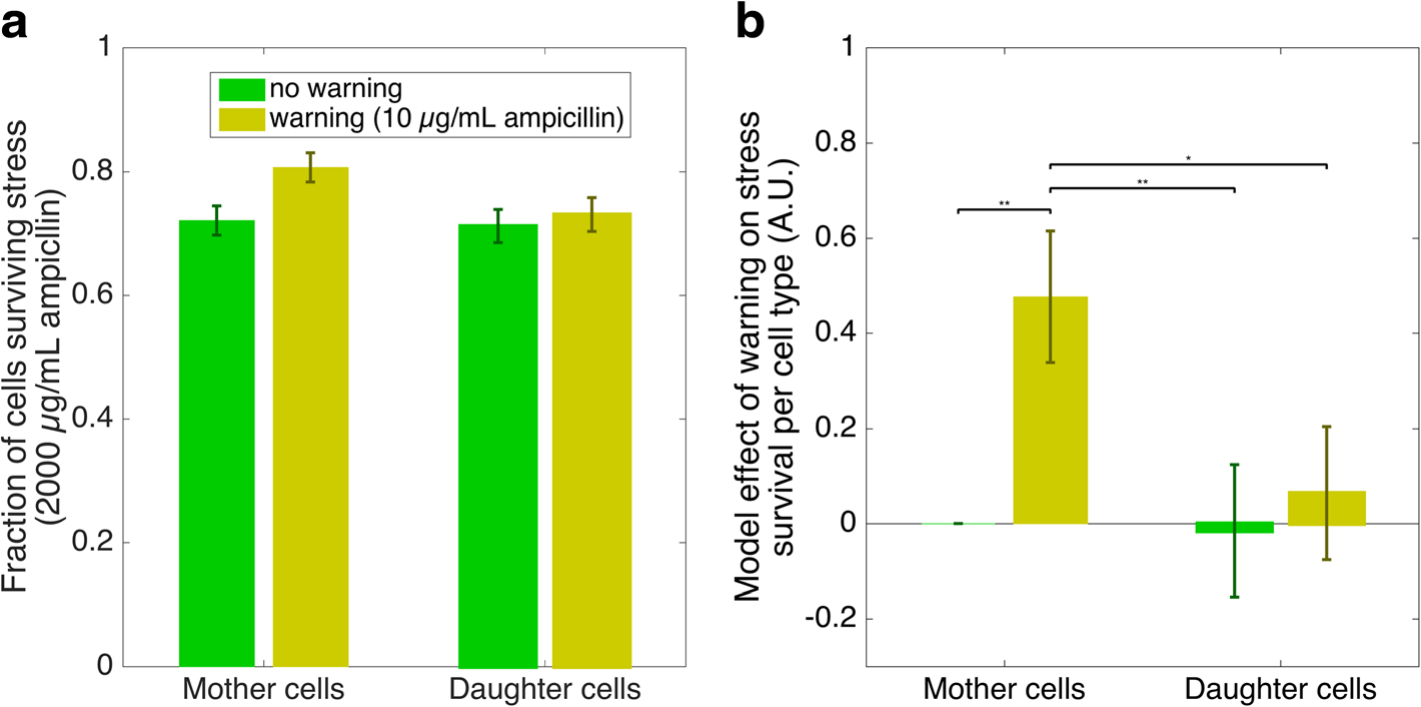
Results from single-cell experiments. A prior warning event increased survival of mother cells, while the warning event had no impact on survival of daughter cells. **a** The two left bars depict the survival probabilities of the 2000 μg/mL ampicillin stress event of stalked mother cells. The mother cells were either directly exposed to the stress event (*green*) or had experienced a 10 μg/mL ampicillin warning event (*yellow*) before the stress event (Fig. 2). The two right bars show the mean survival probabilities of the daughter cells that emerged from divisions of warned (*green*) or unwarned (*yellow*) mother cells in the time period between warning and stress event (Fig. 3: light blue dots emerging from cell divisions between warning and stress event). In total 2’369 cells were analyzed (~90 mother and ~60 daughter cells per replicate channel: 8 channels x 2 conditions x ~150 single cells; see Additional file 7: S4). Bars depict the fraction of cells that survived the stress event (means and standard error of the mean based on 8 replicate channels). The interaction of cell type and warning intensity was significant (*p* < 0.05, see Additional file 7: S5) **b** We fitted a linear model with the factors cell type (mother cell or daughter cell), warning intensity (0 or 10 μg/mL ampicillin) and cell cycle state (see Additional files 3 and 7: S3 and S5). Combinations of factors ‘cell type’ and ‘warning intensity’ were contrasted. We defined unwarned mother cells as the reference condition (left-most bar equals 0). We then compared the effects of each combination (warned/unwarned and mother/daughter) with each other (multiple comparison testing). The effect size compared to the reference is represented by the height of the bar. Top square brackets (⎴) designate significant effects between two conditions (ANOVA post-hoc test. * *p* < 0.05, ** *p* < 0.01, *** *p* < 0.001). The error bars denote standard errors of the mean. The warning had a significant effect on stress survival for the mother cells (*p* = 0.001); and warned mother cells survived significantly better than both warned (*p* = 0.03) and unwarned (*p* = 0.005) daughter cells. The other conditions were not significantly different from each other. In summary the model established that a warning event had a significant effect on survival of mother cells but no significant impact on survival of daughter cells

The analysis (Fig. 4) suggests that survival of the mother cells depended on the warning concentration while survival of daughter cells was not influenced by the prior exposure of their mothers to warning events. We first calculated the fraction of cells surviving a 1-h-exposure to ampicillin for each cell type with and without warning (Fig. 4a). Survival was influenced by cell cycle position and whether or not a cell experienced a prior warning event. The influence of cell cycle position on survival is discussed in the Additional file 3: Figure S3. We fitted a linear model to quantify the effect of the warning on cell survival (Fig. 4b). For the mother cells a prior warning event significantly increased survival of the stress event compared to no prior exposure to ampicillin. In contrast, survival of the daughter cells was not significantly affected by the warning. To summarize this first part, this analysis thus revealed an asymmetry in cellular memory: prior warning events increased the tolerance to stress for mother but not for daughter cells.

This outcome is *consistent* with the hypothesis that we put forward above, that cells only form a cellular memory if this is beneficial, i.e., in situation where past events are informative about future conditions. However, it is important to note that this finding is by no means sufficient to *firmly establish* that this hypothesis is correct. There are many other possible non-evolutionary reasons why swarmer cells would not retain the (unknown) cellular components upon cell division that make their stalked counterpart more tolerant to antibiotics following after the warning, as will be discussed further down. We interpret these results thus not as conclusive evidence for a beneficial asymmetric memory, but rather as an interesting observation that raises new questions. One of the questions that it raises, and that we addressed in the next part, is: do we indeed expect asymmetric memory to evolve in response to a situation where the predictive value of past events differs for the two cell types emerging from division? We used computer simulations to analyze how cellular memory evolves in situation where the predictive value of past events differs for the two cells emerging from division.

### Computational model

Motivated by the experimental results shown above, we used an agent-based simulation to analyze the evolution of cellular memory in simulated bacterial populations. While our main goal here was to investigate the evolution of asymmetric memory, we first asked a simpler question: under what circumstances do we expect evolution of the ability to form a cellular memory? Building on these results, we then analyzed the distribution of memory among asymmetrically dividing cells.

We simulated individual cells with evolvable traits that were genetically encoded. These traits determined the formation of a cellular memory that can provide protection against an external stressor. The level of protection of a given cell was a continuous trait that could change over time, and that determined its survival upon exposure to stress. The current level of protection, and its modulation by the environment, depended on three genetically encoded traits: ‘basal protection’ determined the minimal protection a cell maintained during all times; ‘protection increase’ determined the absolute amount of protection that was added to the current protection level when warning or stress conditions were sensed; ‘protection decrease’ referred to the absolute amount of protection that was subtracted from the current protection level during each time step. If an addition or subtraction of protection to the current level of protection resulted in a protection level higher than 1 or lower than 0, it was corrected in order to ensure that the protection level was always between 0 and 1. When a cell divided, these genetically encoded traits were copied to the two cells emerging from division. At cell division the protection level, which is a phenotypic property of the dividing cell, was divided into two equal parts, and both cells emerging from division received one of these parts (we relaxed this last assumption in the second version of the model, as described below).

In the context of our model, we interpreted the phenotypic trait of increasing the protection level in response to a warning or stress event as the ability to form a cellular memory, and the phenotypic trait of decreasing the protection level under favorable conditions as the ability to erase this memory. The current protection level of a cell then characterized the current state of the cellular memory; it was influenced both by the environment the cell was exposed to in the past as well as by the cell’s genotype that determined how these past events influenced the cellular state. The current protection level determined the probability that a cell would survive a stress event.

**Fig. 5:**
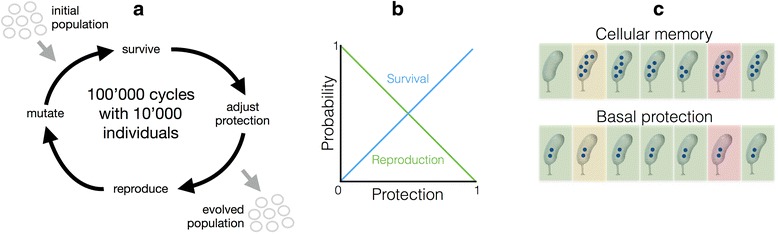
Model description. **a** 10’000 cells were initialized individually by randomly assigning a value from a uniform distribution from 0 to 1 to protection level and each trait (basal protection, protection increase and protection decrease). For every time step the survival of each single cell was evaluated depending on its protection level. Independent of the environmental state 5% of the cells were randomly killed to permit reproduction. In addition, if the current environmental state was ‘stress’, cells were killed dependent on the protection level (phenotype) they were carrying at that specific time point. For surviving cells, the protection level was adapted individually depending on the encoded traits (genotype). Cells were then selected for reproduction with a negative dependency on the protection level. For a small fraction of the population (0.1%) one of the genetic traits was selected randomly and its value was mutated. A list of parameters and pseudocode of the computational model can be found in the Additional file 7: S7. **b** Survival and reproduction probabilities were modeled to be dependent on the current level of protection. Survival of a stress period increased with the level of protection a cell was carrying (blue line). Increasing the level of protection in the current time step led to a decrease in the probability to reproduce, thereby modeled a protection cost. **c** Schematic depiction of two possible genotypes that may arise from this evolution simulation. To illustrate the difference between the two genotypes their behavior is shown in a sequence of time steps (from *left* to *right*, *green* are favorable conditions, *yellow* warning and *red* stress conditions). The number of blue dots within a cell represents the protection level. A cell that had attained a cellular memory (*upper panel*) upregulates protection only when sensing a warning or stress event and maintains the protection (slow decay) despite protection being costly (decreased probability to reproduce). The lower panel shows a cell that has evolved a basal protection where a minimal level of protection is maintained during all time

The environment was modeled as a series of distinct time periods, with each period belonging to one of three types of environmental states: ‘favorable’, ‘warning’ or ‘stress’. A warning period had no negative impact on the cell, but was sometimes (depending on the treatment, see below) preceding a stress period. In repeated simulations we evolved a population of 10’000 individuals for 100’000 time steps (Fig. 5). During ‘stress’ periods mortality of cells was elevated, depending on their protection level. Maintaining a high protection level was costly. The probability of reproduction anti-correlated with the level of protection.

**Fig. 6:**
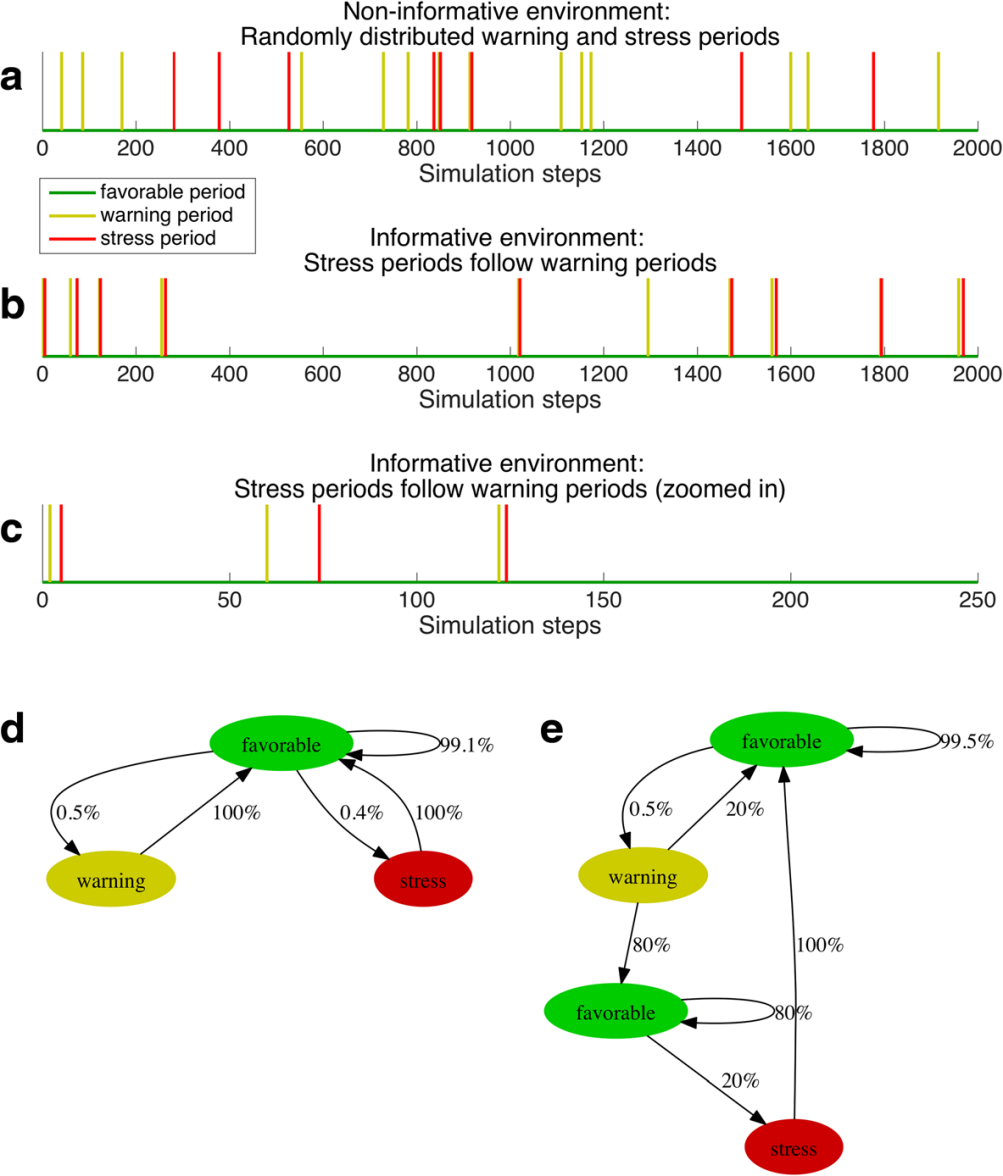
Model of non-informative versus informative environment. We used our model to analyze the evolution of memory in two types of environments denoted informative and non-informative. Both environments were generated with the same overall probabilities of warning (0.5%) and stress (0.4%) periods to occur. Only 2’000 out of 100’000 time steps are shown. In **a** events were distributed randomly without temporal correlation between warning and stress periods. In such an environment the occurrence of a warning period had no informative value to whether a stress period will follow. In **b** a stress period followed a warning event with a probability of 80%. The distance between the warning and the stress event followed an exponential distribution with λ = 0.2 (see Additional file 5: S9.1 and S9.2 for a sensitivity analysis of lambda). Panel **c** shows the first 250 time steps of panel **b**. Panel **d** visualizes how the non-informative environment was constructed. Starting with a favorable period, the probability that the next period was warning was 0.5%, while the probability that the next period was ‘stress’ was 0.4%. Note that ‘warning’ and ‘stress’ periods were independent of each other (except for the fact that stress and warning could not immediately follow each other). Panel **e** visualizes how the environment with informative value was constructed. The informative value lies in the order of periods. A stress period followed a warning period with a probability of 80%

To identify environmental patterns that promote the evolution of the ability to form a cellular memory, we evolved cells in two different types of environments (Fig. 6). In the control environment warning and stress periods were randomly distributed in time and occurred independently of each other (Fig. 6a). Encountering a warning or a stress period had no informative value with respect to which state the environment would attain in the future. In the second environment warning and stress periods were correlated. A warning period often (in four out of five cases) was followed by a stress period (Fig. 6b and c), but usually not immediately so; the time interval between warning and stress period followed an exponential distribution. We expected the ability to form memory to evolve in the informative environment but not in the non-informative environment where the past contained no information about the future and memory was not expected to be advantageous. This first version of our model is related to previous theoretical work that investigated how individual responses to external cues that are indicative of future events evolve in dependence to the reliability of these cues [19, 30–33]. However, in our case cue and future event were separated in time, and their temporal distance was variable. This allowed us to investigate the evolution of *history-dependence* in stress response strategies.

**Fig. 7:**
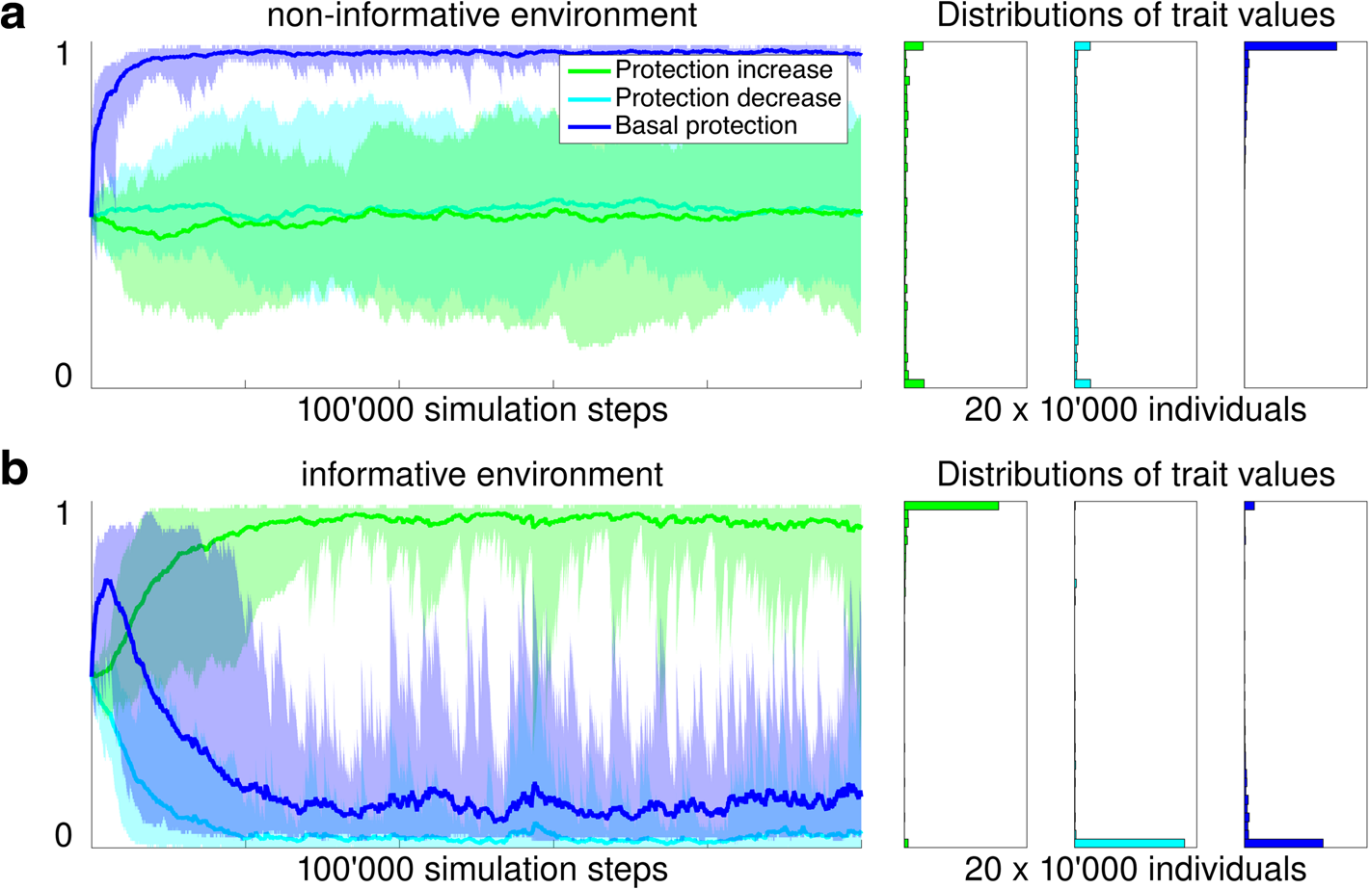
Simulation results of non-informative versus informative environment. The evolution of the three components of protection—protection increase, protection decrease, and basal protection—differed between informative (Fig. 6a) and non-informative environments (Fig. 6b and c). 20 simulations with a population of 10’000 individuals each were run for 100’000 time steps in the two different types of environments. For each time step in the simulation cells survived and reproduced depending on the individual protection level and amount of produced protection. Left panels: The lower and upper boundary of each area corresponds to the minimal and maximal trait value of the 20 simulation runs at each simulation step. The line corresponds to the median trait value. The population means (reported below) were calculated by taking the mean of the 20 population mean trait values of the 10’000 individuals of a particular trait at the end (time point 100’000) of a single simulation. Right panels: For each trait the distribution of the trait values at the end of the simulation (time point 100’000) of all 20 × 10’000 individuals across all 20 simulations is displayed (see Additional file 4: Figure S8.1 for trait distributions of single simulation runs). **a** In an environment where warning and stress periods occurred at random, cells evolved a high basal protection (evolved trait was 0.97, standard error 0.004, left panel: dark blue evolutionary trajectory, right panel: trait values were distributed towards 1). Traits that controlled changes in protection did not evolve to any specific levels, but were random, indicating no selection (protection increase, green: 0.43, standard error 0.038 and protection decrease, light blue: 0.54, standard error 0.036). At the end of the evolutionary process, populations were dominated by types that maintained protection during all times, regardless of history or present environmental conditions (right panel of this figure and schematically represented in Fig. 5c, lower panel). **b** In an environment with correlated warning and stress events, cells evolved a high protection increase (green: 0.94, standard error 0.014) while keeping the basal protection at a low level (dark blue, 0.15, standard error 0.043). This corresponds to up-regulating protection when a warning or stress period was encountered. Protection was not actively down-regulated (light blue: 0.07, standard error 0.042), it gradually decreased at cell division when protection level was evenly distributed to the two cells emerging from division (schematically represented in Fig. 5c, upper panel)

The main outcome of this model was that the ability to form memory evolved in an informative but not in a non-informative environment, in line with our expectations. In non-informative environments cells evolved elevated levels of basal protection (Fig. 7a and Additional file 4: Figure S8.1 for trait distributions of single simulation runs). As upcoming stress events were not predictable, constitutive maintenance of a sufficient level of protection evolved. In contrast, a different genotype evolved in informative environments. Instead of a basal protection, these cells evolved a high protection production rate (Fig. 7b). These types kept protection at a very low level when environmental conditions were favorable. Protection was only increased when a warning or a stress period was encountered. By increasing costly protection during times when conditions were still favorable in anticipation of an upcoming stress event, cells had evolved the ability to make their behavior dependent on past events, or in other words, the ability to form a cellular memory (in form of cellular protection) in response to the warning event, a memory that helped them to better survive stress. These findings are robust to changes in all parameters except to a reduction in the recovery stress switch rate (Additional file 5: Figure S9.1b), a high mutation rate (Additional file 5: Figure S9.4b) and an increase in random death (Additional file 5: Figure S9.6a).

We extended the simulation framework to investigate the evolution of memory in organisms where the two cells emerging from division had different fates. Specifically, we were interested in situations where one cell would remain in the current environment, and the other could migrate and potentially colonize a different environment. We refer to the first cell as the ‘mother’, and the second as the ‘daughter’, as above. We then tested the idea developed above, that such a situation could select for organisms where the mother cell carries a memory, while the daughter cell would not keep a memory. The basis of this hypothesis was that the daughter cell would migrate to a new environment, so that past events in the birth environment were not informative. To address this question with our simulation framework we introduced an additional evolvable trait that we called ‘memory distribution factor’. The memory distribution factor controlled how memory (i.e., the current level of protection, which is a phenotypic trait) was distributed between the mother and the daughter at cell division. A memory distribution factor of 0 corresponded to a situation where the mother kept the entire memory (i.e., the current protection level), while a distribution factor of 1 corresponded to a situation where the daughter cell received the entire memory (i.e., the current protection level). In the previous simulations memory was split equally between these two cells. This corresponded to fixing the memory distribution factor to 0.5. By permitting the memory distribution factor to evolve we expected it to deviate from 0.5 when the past was only informative to the mother but not to the daughter cell.

**Fig. 8:**
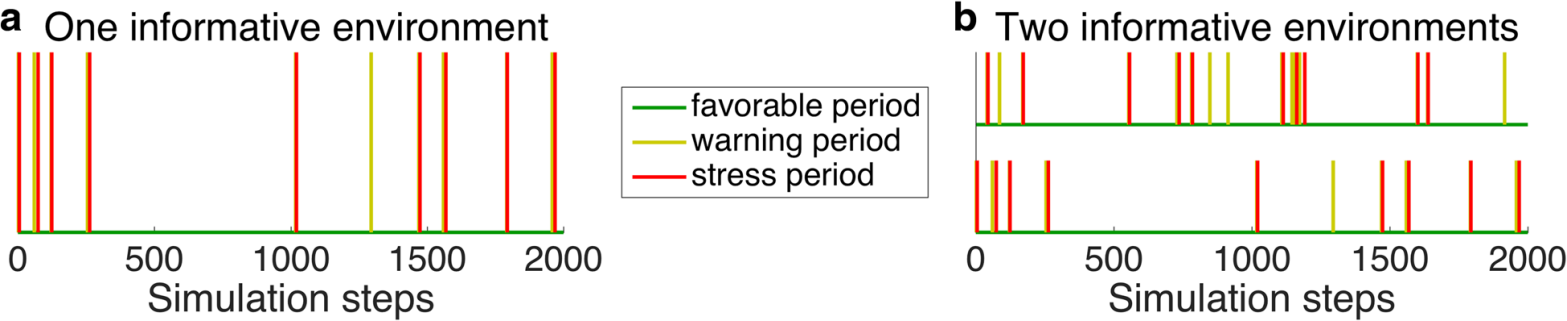
Model of one versus two informative environments. We compared evolution of a trait controlling the distribution of memory, in this case the current level of protection, between the sessile mother and the motile daughter cell following cell division in two different scenarios. Environments were generated according to the state diagram for informative environments (Fig. 6e). Only the first 2’000 of 100’000 time steps are shown. **a** Upon cell division both the mother and the daughter cell remained in the same environment. In this case we expect memory of past events to be equally relevant for both cell types. We thus expected the evolution of a memory distribution factor of 0.5. **b** When cells divided, the motile daughter cell had a 50% chance to either stay in the same environment or to be moved to the other environment (we extended the simulations to 5 environments in Additional file 5: Figure S9.7c). In this case we expected memory to be distributed asymmetrically upon cell division. This can be realized by the evolvable memory distribution factor to deviate from 0.5

To achieve the situation where the mother would stay in the current environment and the daughter would potentially migrate, we carried out simulations using two instead of one environment (Fig. 8b). Each of the two environments was created using the regime for informative environments described above (Fig. 6b, c and e). 80% of the warning events were followed by stress events and the temporal distance between warning and stress event followed an exponential distribution with a mean of 0.2. Importantly, while the different periods were correlated within each environment, they were not correlated *between* environments. Upon cell division the sessile mother cell remained in the same environment, while the motile daughter cell was randomly placed in one of the two environments. That way, the mother cell always stayed in the same environment, while the daughter cell had a 50% chance to leave the current environment. Once the daughter cell was placed in one of the two environments it differentiated to a mother cell and initiated reproduction.

We expected that this situation would select for organisms where memory, i.e. protection, would be distributed asymmetrically at cell division: for the mother, past events were informative about the future, and thus the mother was expected to maintain memory. For the daughter, past events were less predictive about the future (since it only stays in the same environment in 50% of the cases), and we thus expected that it would keep less memory. In the context of our model, such asymmetric distribution of memory would manifest as a memory distribution factor smaller than 0.5. As a control, we ran the simulations with the same evolvable traits in a single environment where both cell types emerging from a cell division were staying in the same environment (Fig. 8a).

**Fig. 9:**
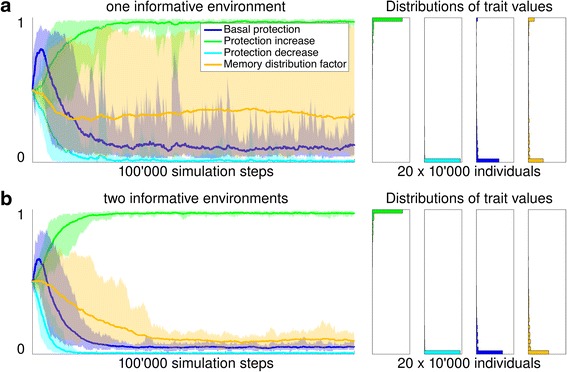
Simulation results of one versus two informative environments. Migration of daughter cells into new environments promoted the evolution of asymmetric memory to be passed on to the mother cells at cell division. 20 simulations were run for 100’000 time steps with 10’000 cells in total for each of the two environment types described in Fig. 8. Cells were initialized with randomly generated trait values for the four components of protection. For each time step in the simulation cells survived and reproduced depending on the individual protection level. Left panels: The lower and upper boundary of each area corresponds to the minimal and maximal trait value of the 20 simulation runs at each simulation step. The line corresponds to the median trait value. Right panels: For each trait the distributions of the trait values at the end (time point 100’000) of the simulation of the 20 × 10’000 individuals across all 20 simulations is displayed. **a** When all cells were kept in a single environment, the memory distribution factor trait of the 20 simulations with different initial values did not converge (orange, 0.43, standard error: 0.08), but evolved to an extreme (close to 0 or close to 1) in most of the replicated populations (see Additional file 4: Figure S8.2 left panels for trait distributions of single simulation runs). **b** When some of the daughter cells migrated to a different environment, asymmetric segregation of cellular protection to the mother cells evolved (orange trajectories converged to a low value; mean: 0.092, standard error: 0.007)

When populations of cells were evolved in two environments (Fig. 8b), we observed evolution of asymmetric memory distribution upon cell division, as we hypothesized (*orange trajectory* in Fig. 9b and Additional file 4: Figure S8.2 for trait distributions of single simulation runs). Upon cell division, about 90% of the memory, i.e. protection, segregated to the mother cell, and only about 10% to the motile daughter cell. While this result corresponds to the hypothesis put forward above (that the mother cell will keep a cellular memory) it can also be looked at from a different perspective: that daughter cells emerge from division without any memory about past events in their birth environment. One way to interpret this is that the daughters actively ‘forget’ past events that are not informative for the future conditions that they will experience. These findings are robust to changes in all parameters except to a reduction in the recovery stress switch rate (Additional file 5: Figure S9.1c and d) and a high mutation rate (Additional file 5: Figure S9.4d).

In the control environment, where both mother and daughter remained in same environment, we found an unexpected result: while there was no consistent distribution of protection (Fig. 9a, *orange*) to either mother or daughter *across* the 20 replicate populations (which is to be expected because mother and daughters are equivalent in this setting), *within* most of the 20 replicate populations there was a clear tendency for protection to be either passed on to the mother cells (memory distribution factor close to 0) or to the daughter cells (memory distribution factor close to 1). This means that most of these 20 replicated populations evolved a type of asymmetric memory where cellular protection was passed on to one of the two cells emerging from division, rather than distributed to both types equally (see Additional file 4: S8.2 left panels, for memory distribution factor trait distributions, *orange*, per single simulation). We note that this outcome is not equivalent to the situation with two environments, where cellular protection consistently is passed on to the mother cell that stays in the same environment (*orange*, Fig. 9b and Additional file 4: S8.2 right panels), consistent with the idea put forward above. We see a possible interpretations for this asymmetry observed in the control populations: distributing protection asymmetrically leads to variation between the two cells emerging from division, and this could increase the long-term growth rate of these types by decreasing the temporal variation in survival. This decrease in temporal variation results from the fact that through this asymmetry a fraction of the individuals are well prepared for stress, while others are well prepared for favorable conditions. This effect is known as bet-hedging [34–36].

These simulations indicate that memory is expected to evolve in an informative environment (Fig. 7b) and that it is distributed asymmetrically to the cell types for which past conditions are informative about the future (Fig. 9b). Asymmetric segregation of cellular memory between the two cells emerging from division can thus be indicative of an adaptive role of cellular memory; this is because asymmetric segregation can be a manifestation of differences between cells in how informative events are to predict future events. However, it is important to note that we observed that asymmetric memory could also evolve for other reasons (see Fig. 9a), potentially because it can reduce temporal variation in survival through a bet-hedging mechanism.

While using these agent-based simulations to explore the evolution of cellular memory in different environments can be useful for testing verbal arguments and guiding experimental investigations, we want to emphasize that the behavior observed in biological systems is more complex than what is captured by our model. We tried to keep the number of parameters as low as possible to and make sensible choices (also see a discussion on the sensitivity on parameter values in Additional file 5: S9), to increase the probability of finding general patterns.

## Conclusions

In conclusion, the asymmetry that we observed between mother and daughter cells regarding the influence of past events on future behavior is consistent with the view that cellular memory might be shaped by natural selection to increase survival and reproduction in the face of environmental fluctuations. Our computer simulations indicate that the ability to form a cellular memory can evolve in situations where the past is informative with respect to future events, and that asymmetric memory is expected to evolve if the past is predictive for one cell type but not for the other. Asymmetric cellular memory might indeed be a possible explanation of the experimental results, in the sense that a previous ‘warning’ increased survival during antibiotic exposure for the sessile mother but not for the motile daughter cells. However, we cannot rule out alternative hypotheses. *C. crescentus* stalked cells and swarmer cells differ in behavior and motility, and this results in differences in a large number of cellular features, including proteome composition [37], transcriptional [38, 39] and translational activity [40] and second-messenger signaling [41]. It is well possible that the relative small asymmetry in cellular memory that we observed is an unselected consequence of these cellular differences, rather than a consequence of natural selection acting differently on history-dependent behavior of swarmer and stalked cells.

While we thus cannot currently exclude alternative explanations for this finding, these experimental and theoretical results raise the interesting question whether microorganisms might evolve specificity in the cellular memory, in the sense that they store information about past events specifically in cases where these past events are informative with respect to future conditions. Asymmetrically dividing cells are an interesting model system to further investigate the nature and function of bacterial memory.

## Methods

The microfluidic devices used were adapted from the ‘mother machine’ design [42]. Masks for photolithography were ordered from ML&C GmbH, Jena, Germany. Two-step photolithography was used to obtain silicon wafers. Silicone elastomers (Sylgard 184 Silicone Elastomer Kit, Dow Corning) were prepared by mixing the two components in a ratio of 10:1, poured on the dust-free wafer, de-aired in a desiccator to eliminate air bubbles, and incubated overnight at 80 °C for curing. Polydimethylsiloxane elastomer (PDMS) chips of approximately 2.5 cm × 4 cm were cut out around the structures of the wafer. Each PDMS chip featured 8 separate channels (we did not make use of the narrow side channels of the ‘mother machine’ design because *C. crescentus* attaches to the glass slide naturally)*.* This enabled us to test 8 conditions in one experiment (Fig. 1b). The channels were 22 μm deep and 100 μm wide. Holes for medium supply and outlet were punched using a 20 G needle (1.2 mm × 40 mm) that was modified by breaking off the beveled tip and sharpening the edges of the now straight tip. The surface residues on the PDMS chips and on the round (50 mm diameter) glass coverslips (Menzel-Gläser, Braunschweig, Germany) were chemically activated by treating both surfaces for 6 min in a UV-Ozone cleaner (Novascan PSD-UV). The PDMS chips were then placed on the glass cover slips, the exposed sides facing each other, and heated at 100 °C for 1 h to ensure binding. Before the experiment, the chips were rinsed with PYE medium (see below) with a flow rate of 3.5 mL/h until the channels were filled. This was done using 1 mL syringes (Codan/Once Primo, Huberlab) with a single-syringe pump (NE-300, NewEra Pump Systems).

We constructed a transcriptional fusion of the *blaA* gene with *egfp* (green fluorescent protein). The chromosomal setup suggests that *blaA* (CC2139) is part of an operon with three other genes (CC2141, CC2140, CC2138) [22]. We amplified the promoter region of CC2141 and fused it with e*gfp* (green fluorescent protein). The amplified region was inserted into *pMR10* background. The resulting plasmid was transformed into wildtype *C. crescentus* CB15 [28], ATCC 19089. GFP expression from this plasmid was used to assess induction of *blaA (see* Additional file 6: Figure S6)*.* The GFP signal was not used for data analysis except for the findings reported in Additional file 6: Figure S6.

Bacteria were grown overnight in culture tubes (100 mm × 16 mm PP reaction tube, Sarstedt, Nümbrecht, Germany) in 5 mL PYE complex medium [43] shaking at 220 rpm at 30 °C, and then diluted 1:10’000 in 25 mL PYE in a 50 mL Falcon tube to obtain exponentially growing cells. When the culture reached an OD600 of 0.2, 12.5 mL of cells were centrifuged for 3 min at 4600 × g in a 15 mL Falcon tube. Supernatant was discarded and cells were resuspended in the remaining 500 μL and loaded into a 1 mL syringe (Codan/Once Primo, Huberlab). The cells were then pumped into the channel of the microfluidic chip for 1 min at a rate of 3.5 mL/h using a single-syringe pump (NE-300, NewEra Pump Systems). The cells were incubated in the chip for 20 min at 30 ° C. During that time swarmer cells attached to the surfaces of the main channel of the chip (Fig. 3) and later differentiated to stalked cells and started to divide.

For the experiment two pumps (NE-1800, NewEra Pump Systems) with 8 syringes in parallel were used. This ensured constant growth conditions, keeping the sessile cells in exponential phase and preventing the formation of biofilm. 50 mL syringes (Pic Solution) were loaded with 50 mL PYE and used for the non-stress periods. For the ampicillin warning and stress events, 10 mL syringes (Soft-Ject) were loaded with 2 mL of PYE + ampicillin (2, 10 or 2000 μL/mL depending on the condition applied). Tubing (Microbore Tygon X74HL, ID 0.76 mm, OD 2.29 mm, Fisher Scientific) was connected to the syringes using 20G needles (0.9 mm × 70 mm, Huberlab). Smaller tubing (PTFE, ID 0.3 mm, OD 0.76 mm, Fisher Scientific) was then inserted into the bigger tubing (Tygon S54HL), and directly connected to the inlet hole in the PDMS chip. Medium change was performed by disconnecting and reconnecting the tubing from the PYE to PYE + ampicillin. Pumping speed during the experiments was set to 2 mL/h.

Microscopy was performed using an Olympus IX81 inverted microscope system with automated stage, shutters, and laser based autofocus system. Several positions were monitored in parallel on the same device, and phase contrast images of every position were taken every 5 min. Images were acquired using an UPLFLN40x phase contrast objective (Olympus) and a cooled CCD camera (Olympus XM10). For image acquisition, the Xcellence Pro software package (Olympus, Version 1.2) was used. To keep temperature at 30 ° C the microscope was placed in an incubated box (Life Imaging Services, Reinach, Switzerland). Fluorescence images were acquired using a 120 W mercury short arc lamp (Xcite 120PC Q) and the U-N49002 EGFP filter set (450–490 nm ex/500–550 em/495 dichroic mirror, Chroma).

Images were analyzed with ImageJ. A plugin (*CellCounter*, Kurt de Vos, University of Sheffield, http://rsb.info.nih.gov/ij/plugins/cell-counter.html) was adapted such that cells could be marked across all time frames. Division frames of each cell were stored in a XML file upon visual inspection. Simulations were programmed in C++. Matlab and R were used for data analysis. The complete code of the computational model can be obtained from the corresponding author upon request.

## Additional files

Additional file 1: Figure S1. In average 37% of the daughter cells that emerged from cell divisions attached to the glass slide. The dark blue line shows the total number of divisions observed for the original population of stalked mother cells per 5-min time interval. The light blue line shows how many of these newly emerged daughter cells attached to the glass slide. The newly emerged daughter cells that did not attach were washed out. (PNG 324 kb)

Additional file 2: Figure S2. The warning event had no detectable influence on cell division timing, while the stress event delayed cell divisions of mother and daughter cells and led to cell cycle synchronization. (A) The number of cell divisions per 5-min time interval during the course of the experiment is shown for unwarned cells (green trajectory) and cells that were exposed to a warning event (yellow). The favorable/warning period is represented in grey (0, or 10 µg/mL ampicillin during 2 h), the stress period in red (2000 µg/mL ampicillin during 1 h) areas respectively. In different colors the mean number of cell divisions per 5-min interval for each warning condition event is shown (*N* = 763 and 742 for exposure to 0 and 10 µg/mL ampicillin during the warning event). (B) The influence of the warning and stress event on the interdivision time (time since last division before event + time to next division after event) was analyzed. The area of the grey circles corresponds to the number of cells (smallest circle corresponds to 1 cell, largest circle corresponds to 48 cells) found in the experimental data. In color the median is shown for unwarned (green) and warned (yellow) cells. The warning event had no detectable effect on the interdivision time (left panel, overlapping green and yellow lines following a diagonal). In contrast, the stress event had a clear effect on the interdivision time (middle and right panel): Mother cells for which the last division had been less than 50 min ago divided only after around 150 min after the onset of the stress event. The first division of a daughter cell takes longer due to differentiation into a stalked mother cell. This delay can be observed when comparing the middle and the right panel. (PNG 868 kb)

Additional file 3: For each cell, cell cycle position was estimated at the time-point when cells were exposed to the stress event (2000 µg/mL ampicillin for 1 h). Since the time period between warning and stress event exceeds the time to the first division of daughter cells, some of the daughter cells in our analysis had already divided. These daughter cells, while being daughters of mothers that had experienced the warning event, are staked cells that had already divided once. To correct for the cell cycle state we therefore needed to correct the daughter cells that had already divided differently from the daughter cells that had not yet divided. For the daughter cells that had not yet divided we used a cell-cycle position correction that accounted for their longer interdivision time (in our system the interdivision time of a cell that emerges as a swarmer and then stays in the microfluidic device is about 15–20 min longer than the interdivision time of the stalked cell cycle). The cells that had already divided were corrected the same way as the mother cells that were born before the warning event since their cell cycle timing is the same. For both types of cells, cell cycle position was approximated by the time that had passed since the last division. **Figure S3**. Survival of the stress event was dependent on cell cycle position. (A and B) For the number of cells that had already divided before (A) and cells that were in the process of dividing for the first time (B) cell cycle position at the time of onset of stress is depicted. (C and D) Survival per cell cycle position and cell type is shown in fractions and was modeled with a second-degree polynomial. For the model the filled bars in panel A and B were used (cell cycle position 5–70 for mother cells and 5–90 for daughter cells). (PNG 510 kb)

Additional file 4: In the following plots the distribution of trait values at the end of a simulation of 10’000 individuals are shown. Each row corresponds to a single simulation run, each column to a trait. The title marks the type of environment that was used (see Fig. 6-9). Finding subpopulations with high basal protection in informative environments (Figure 8.1 right panel: blue bars with high basal protection) possibly indicates the evolution of a bet-hedging strategy. **Figure S8.1.** Trait distributions from single simulations in non-informative and informative environments. Trait (columns) distributions of the 20 simulation runs (rows) in a non-informative environment (*left*, see Fig. 7a) and 20 simulations runs in an informative environments (*right*, see Fig. 7b). **Figure S8.2** Trait distributions from single simulations in one informative and two informative environments. Trait (columns) distributions of the 20 simulation runs (rows) in one informative environment (*left*, see Fig. 9a) and 20 simulations runs in two informative environments (*right*, see Fig. 9b). (ZIP 21 kb)

Additional file 5: To assess the sensitivity of the simulation outcomes to varying simulation parameters, we changed a single simulation parameter at a time and rerun the simulations shown in Fig. 7 and 9. Since we did not vary all of the parameters and did not change more than one parameter at a time, this is not exhaustive. The following table lists the parameter values used in the simulations in Fig. 5 and 7 in the column Default. For each parameter we chose a lower and a higher value to rerun the simulation (columns Lower and Higher). See description and usage of the parameters in supplementary material S7. The following figures show the results from simulations where single parameters were changed compared to the reference parameters used in Figs. 7 and 9. **Figure S9.1**: lambda = 0.1. **Figure S9.2**: lambda = 0.4. **Figure S9.3**: mutRate = 0.0001. **Figure S9.4**: mutRate = 0.01. **Figure S9.5**: rndKill = 0.01. **Figure S9.6**: rndKill = 0.1. **Figure S9.7**: daughtersAlwaysLeave, daughtersAlwaysStay and numEnv = 5. Lowering the rate to switch from recovery phase to stress phase to 0.1 led to the evolution of a genotype with a high basal protection as was observed with a lambda of 0.2 (compare **Figure S9.1a** to **Fig. 7a**). But we did no longer observe the evolution of a memory genotype (compare **Figure S9.1b** to Fig. 7b and **Figure S9.1c** to Fig. 9a). Interestingly a phenotype that segregated cellular protection only to one of the two cells emerging from division still did evolve in the case of two environments (**Figure S9.1d**). The simulation trajectories were comparable to the reference when lambda was increased from 0.2 of 0.4 (compare Figs. 7 and 9 to **Figure S9.2**). Qualitatively we observed the same simulation outcome when decreasing the mutation rate from 0.01 to 0.0001, although a slower convergence was observed (compare Figs. 7 and 9 to **Figure S9.3**). Simulation results that were run with an increased mutation rate (0.1) diverged from what we observed in the reference simulations (compare Figs. 7 and 9 to **Figure S9.4**). Note the bimodal distributions of both basal protection and the memory distribution factor in **Figure S9.4c** and **S9.4d**. Decreasing the fraction of individuals that are killed randomly in each simulation round from 0.05 to 0.01 led to the evolution of a basal protection genotype independent of information content and number of environments (**Figure S9.5**). In these simulations the carrying capacity of the population (10’000 individuals) was almost always exhausted, there was not enough ‘room’ for evolutionary mechanisms in the 100’000 time steps. When increasing the killing rate to 0.1 the mean basal protection that evolved in a random environment was significantly lower compared to the reference (compare **Figure S9.6a** to Fig. 7a). A high population turnover favors a genotype with an intermediate basal protection to increase probability of reproduction. The simulation results observed when increasing random killing of individuals from 0.05 to 0.1 were comparable to the reference (compare panel S9.6b, c and d to Figs. 7b, 9a and b). A set of simulations was run with the same parameters as shown in Fig. 9b, but daughter cells were not randomly moved to one of the two environments. Instead, the daughter cells were always moved to the environment where they were not ‘born’. This had an impact on the evolution of the memory distribution factor (mean 0.07 in **Figure S9.7a** versus 0.03 in the reference environment Fig. 9b). As expected we did not observe asymmetric memory when simulating two informative environments, where daughter cells were forced to stay in the environment they were born (**Figure S9.7b**). Increasing the number of informative environments from 2 to 5 had no noticeable impact on the evolution results (**Figure S9.7c**). (ZIP 5.56 mb)

Additional file 6: Figure S6. It has been reported that the *blaA* (CC2139) gene is a major contributor to ampicillin resistance in *C. crescentus* [22]. We measured expression of *blaA* using a transcriptional reporter (see Methods). GFP (green fluorescent protein) intensity, a proxy for transcriptional activity of *blaA,* was measured before (t1) and after (t2) the warning event (0 or 10 µg/mL ampicillin for 2 h). After background correction the intensity level at t2 was subtracted from the intensity level at t1. (A) The mean of the differences is represented by the bars (error bars denote standard errors of the mean). For both conditions the intensity levels at t1 was compared to the intensity levels at t2 using a paired *t*-test statistic (*N* = 117 and 119 for 0 and 10 µg/mL ampicillin exposure). When cells were exposed to 10 µg/mL ampicillin for 2 h (yellow), the increase of the measured GFP intensity was significant (*p* < 0.01), while for the unwarned cells (green) no significant changes were observed (*p* = 0.3). (B) Numbers of surviving and dead cells are reported for small intervals of GFP intensity values (20 bins from -1.5 to 2.5 with size 0.2). No statistically significant association of GFP levels with survival after stress (logistic regression *p* = 0.68) could be established. (PNG 272 kb)

Additional file 7: Additional tables and listings. (PDF 143 kb)
