# Supplementary figures and images for: Asymmetric cellular memory in bacteria exposed to antibiotics
Source: BMC Evol Biol. 2017 Mar 9;17:73. doi: 10.1186/s12862-017-0884-4 (PMC5343395; doi:10.1186/s12862-017-0884-4)

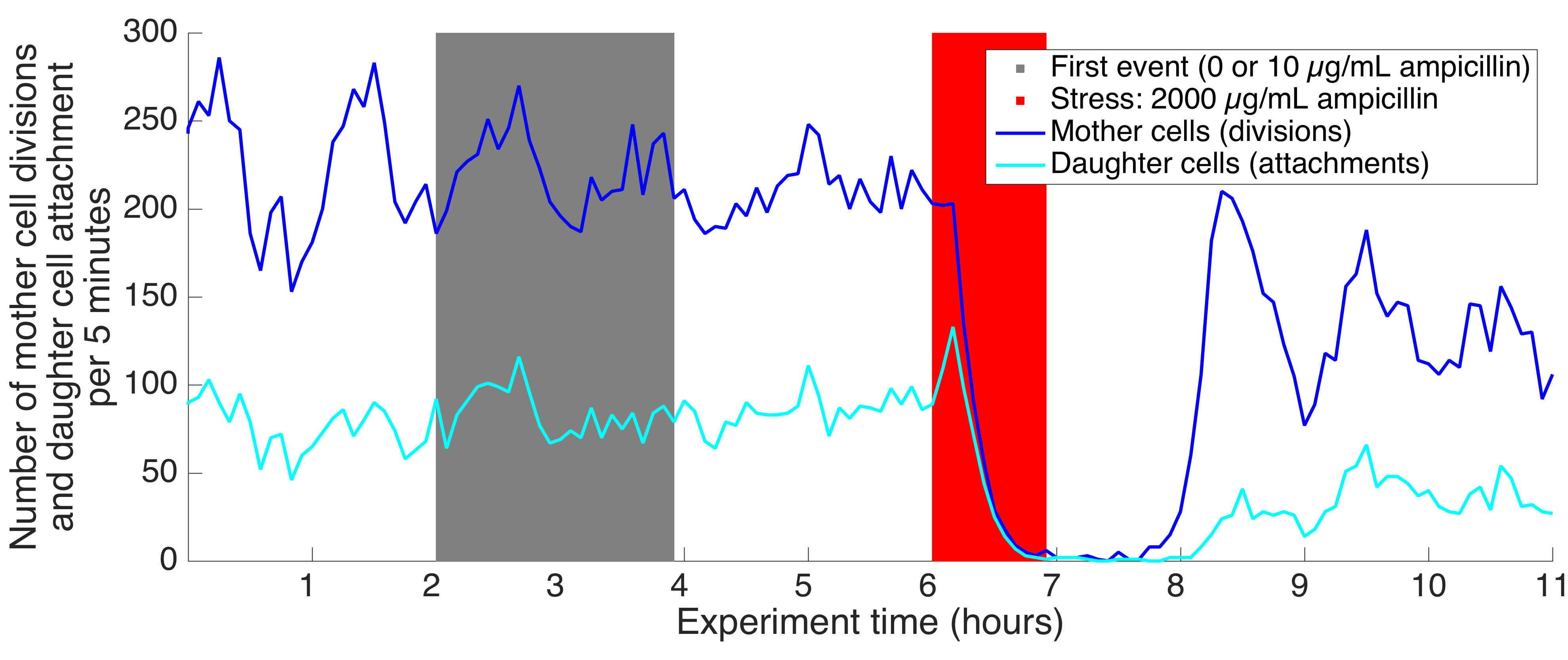

Supplement: Supplementary file 1 — In average 37% of the daughter cells that emerged from cell divisions attached to the glass slide. The dark blue line shows the total number of divisions observed for the original population of stalked mother cells per 5-min time interval. The light blue line shows how many of these newly emerged daughter cells attached to the glass slide. The newly emerged daughter cells that did not attach were washed out. (PNG 324 kb) [file 12862_2017_884_MOESM1_ESM.png]

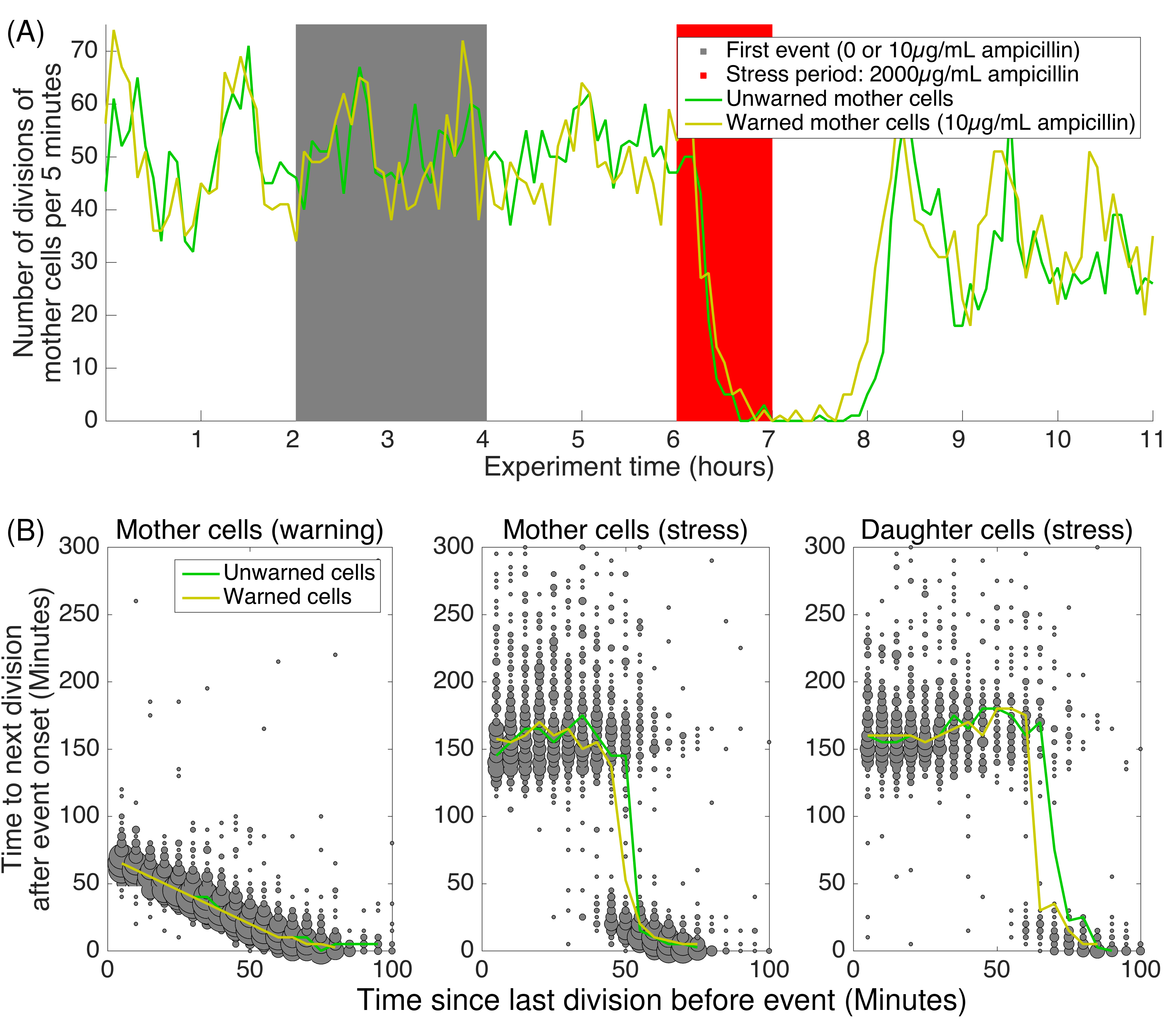

Supplement: Supplementary file 2 — The warning event had no detectable influence on cell division timing, while the stress event delayed cell divisions of mother and daughter cells and led to cell cycle synchronization. (A) The number of cell divisions per 5-min time interval during the course of the experiment is shown for unwarned cells (green trajectory) and cells that were exposed to a warning event (yellow). The favorable/warning period is represented in grey (0, or 10 µg/mL ampicillin during 2 h), the stress period in red (2000 µg/mL ampicillin during 1 h) areas respectively. In different colors the mean number of cell divisions per 5-min interval for each warning condition event is shown (N = 763 and 742 for exposure to 0 and 10 µg/mL ampicillin during the warning event). (B) The influence of the warning and stress event on the interdivision time (time since last division before event + time to next division after event) was analyzed. The area of the grey circles corresponds to the number of cells (smallest circle corresponds to 1 cell, largest circle corresponds to 48 cells) found in the experimental data. In color the median is shown for unwarned (green) and warned (yellow) cells. The warning event had no detectable effect on the interdivision time (left panel, overlapping green and yellow lines following a diagonal). In contrast, the stress event had a clear effect on the interdivision time (middle and right panel): Mother cells for which the last division had been less than 50 min ago divided only after around 150 min after the onset of the stress event. The first division of a daughter cell takes longer due to differentiation into a stalked mother cell. This delay can be observed when comparing the middle and the right panel. (PNG 868 kb) [file 12862_2017_884_MOESM2_ESM.png]

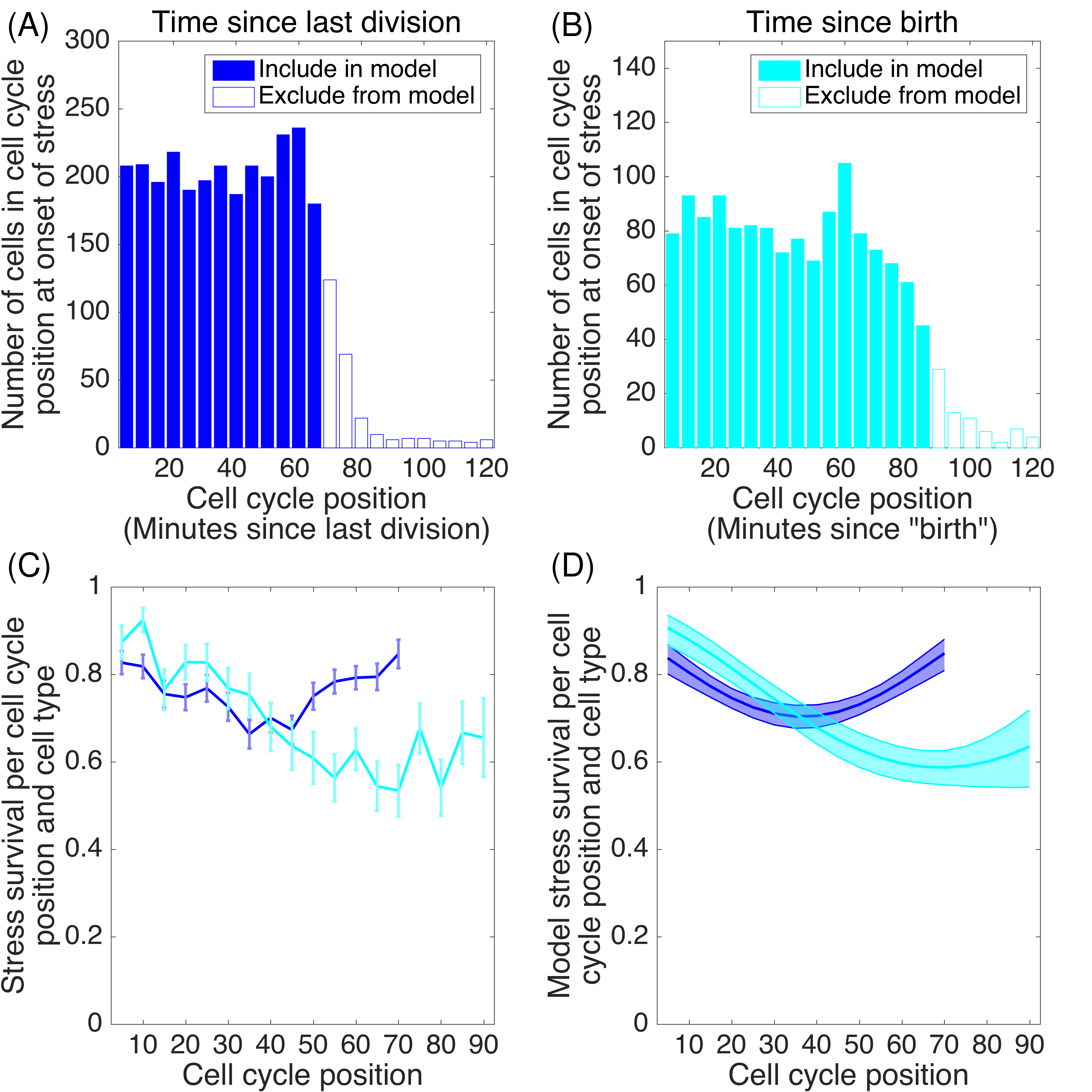

Supplement: Supplementary file 3 — For each cell, cell cycle position was estimated at the time-point when cells were exposed to the stress event (2000 µg/mL ampicillin for 1 h). Since the time period between warning and stress event exceeds the time to the first division of daughter cells, some of the daughter cells in our analysis had already divided. These daughter cells, while being daughters of mothers that had experienced the warning event, are staked cells that had already divided once. To correct for the cell cycle state we therefore needed to correct the daughter cells that had already divided differently from the daughter cells that had not yet divided. For the daughter cells that had not yet divided we used a cell-cycle position correction that accounted for their longer interdivision time (in our system the interdivision time of a cell that emerges as a swarmer and then stays in the microfluidic device is about 15–20 min longer than the interdivision time of the stalked cell cycle). The cells that had already divided were corrected the same way as the mother cells that were born before the warning event since their cell cycle timing is the same. For both types of cells, cell cycle position was approximated by the time that had passed since the last division. Figure S3. Survival of the stress event was dependent on cell cycle position. (A and B) For the number of cells that had already divided before (A) and cells that were in the process of dividing for the first time (B) cell cycle position at the time of onset of stress is depicted. (C and D) Survival per cell cycle position and cell type is shown in fractions and was modeled with a second-degree polynomial. For the model the filled bars in panel A and B were used (cell cycle position 5–70 for mother cells and 5–90 for daughter cells). (PNG 510 kb) [file 12862_2017_884_MOESM3_ESM.png]

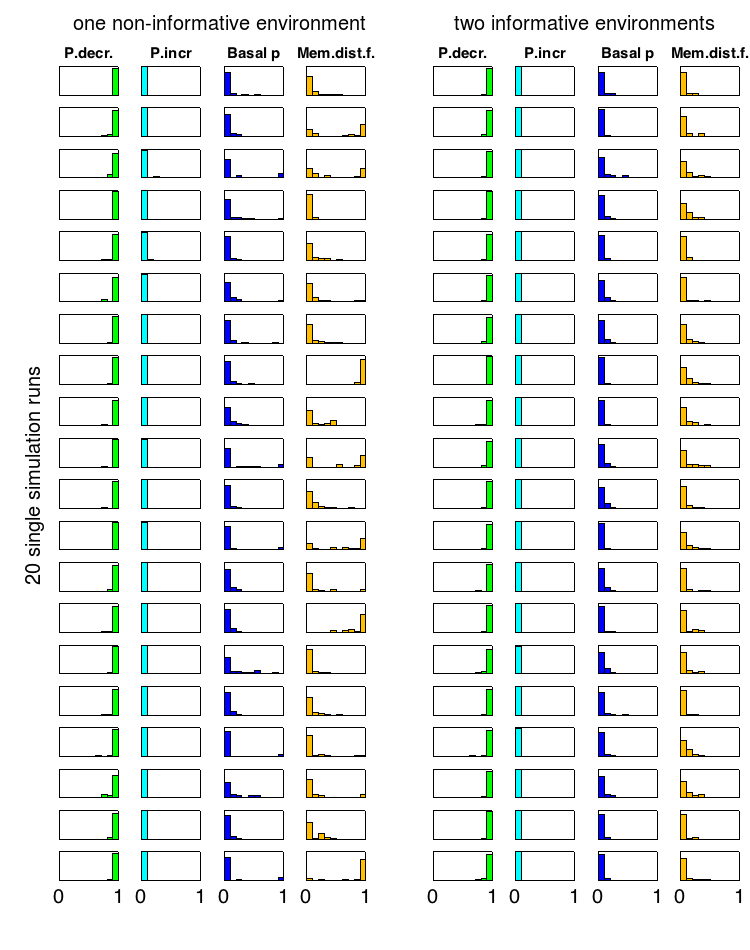

Supplement: Supplementary file 4 — In the following plots the distribution of trait values at the end of a simulation of 10’000 individuals are shown. Each row corresponds to a single simulation run, each column to a trait. The title marks the type of environment that was used (see Fig. 6-9). Finding subpopulations with high basal protection in informative environments (Figure 8.1 right panel: blue bars with high basal protection) possibly indicates the evolution of a bet-hedging strategy. Figure S8.1. Trait distributions from single simulations in non-informative and informative environments. Trait (columns) distributions of the 20 simulation runs (rows) in a non-informative environment (left, see Fig. 7a) and 20 simulations runs in an informative environments (right, see Fig. 7b). Figure S8.2 Trait distributions from single simulations in one informative and two informative environments. Trait (columns) distributions of the 20 simulation runs (rows) in one informative environment (left, see Fig. 9a) and 20 simulations runs in two informative environments (right, see Fig. 9b). (ZIP 21 kb) [file 12862_2017_884_MOESM4_ESM.zip › FigureS8_2.png]

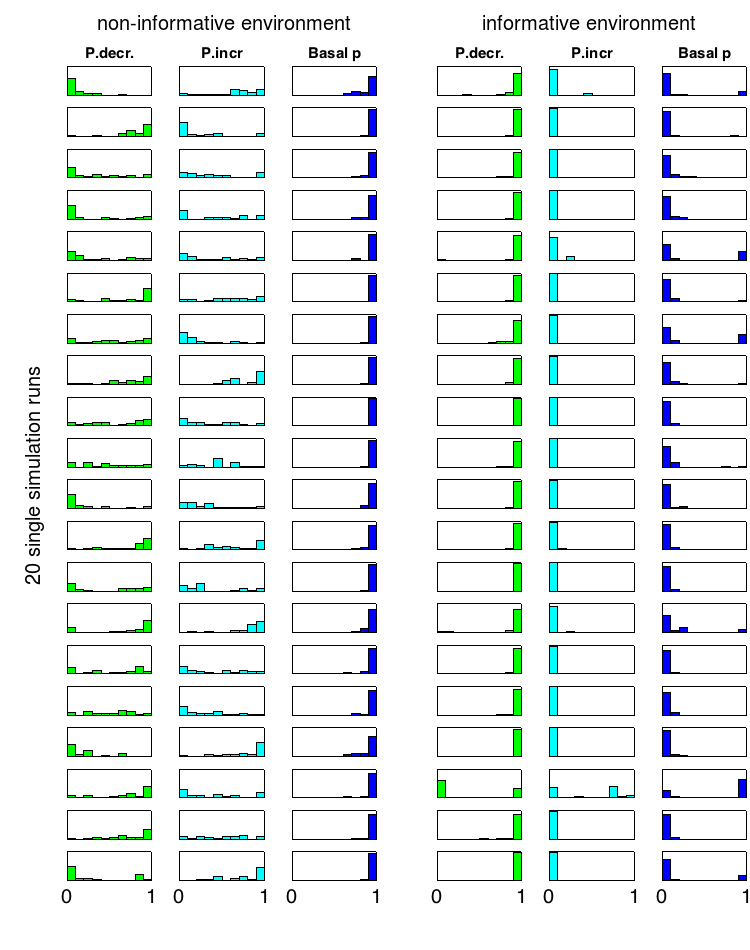

Supplement: Supplementary file 4 — In the following plots the distribution of trait values at the end of a simulation of 10’000 individuals are shown. Each row corresponds to a single simulation run, each column to a trait. The title marks the type of environment that was used (see Fig. 6-9). Finding subpopulations with high basal protection in informative environments (Figure 8.1 right panel: blue bars with high basal protection) possibly indicates the evolution of a bet-hedging strategy. Figure S8.1. Trait distributions from single simulations in non-informative and informative environments. Trait (columns) distributions of the 20 simulation runs (rows) in a non-informative environment (left, see Fig. 7a) and 20 simulations runs in an informative environments (right, see Fig. 7b). Figure S8.2 Trait distributions from single simulations in one informative and two informative environments. Trait (columns) distributions of the 20 simulation runs (rows) in one informative environment (left, see Fig. 9a) and 20 simulations runs in two informative environments (right, see Fig. 9b). (ZIP 21 kb) [file 12862_2017_884_MOESM4_ESM.zip › FigureS8_1.png]

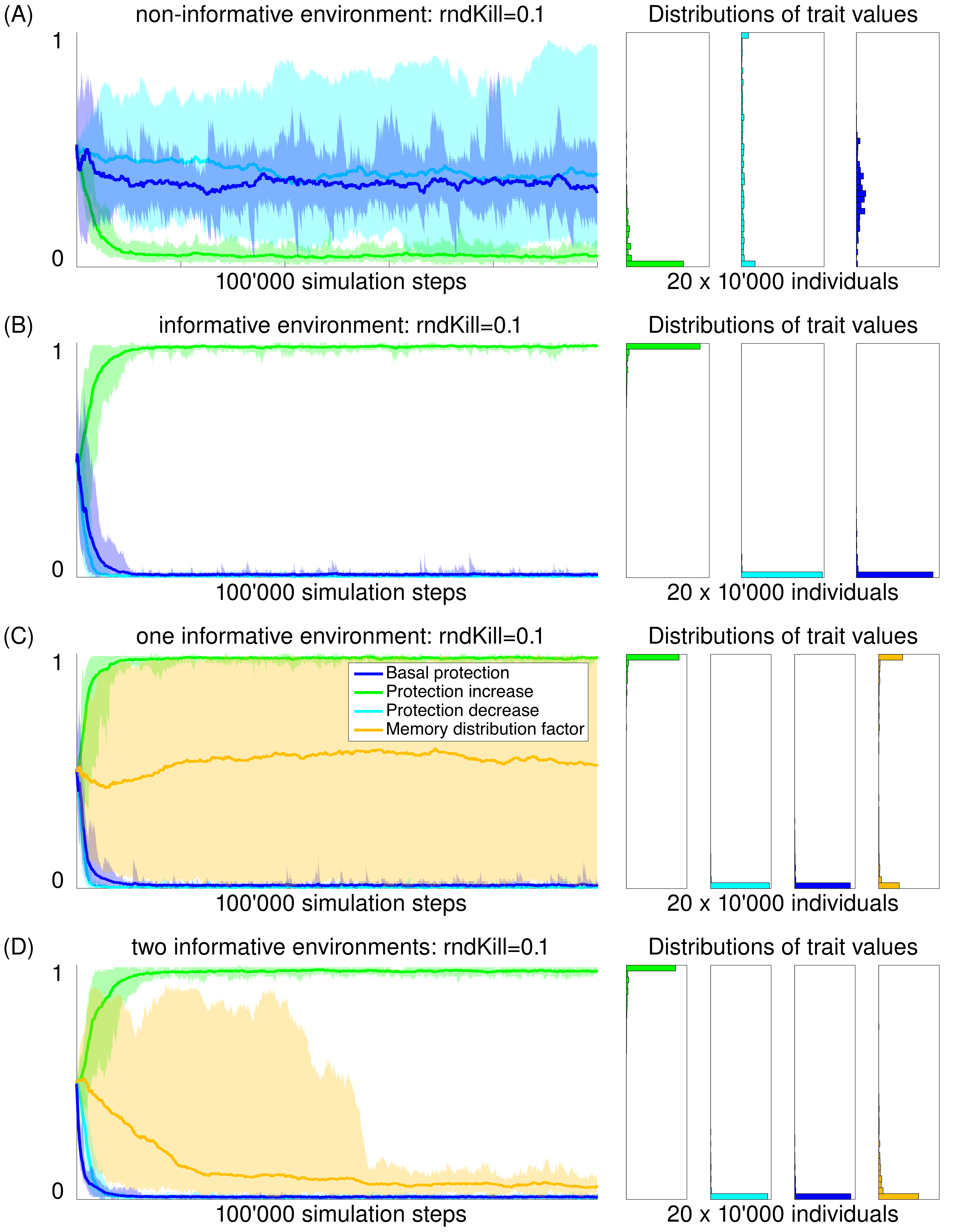

Supplement: Supplementary file 5 — To assess the sensitivity of the simulation outcomes to varying simulation parameters, we changed a single simulation parameter at a time and rerun the simulations shown in Fig. 7 and 9. Since we did not vary all of the parameters and did not change more than one parameter at a time, this is not exhaustive. The following table lists the parameter values used in the simulations in Fig. 5 and 7 in the column Default. For each parameter we chose a lower and a higher value to rerun the simulation (columns Lower and Higher). See description and usage of the parameters in supplementary material S7. The following figures show the results from simulations where single parameters were changed compared to the reference parameters used in Figs. 7 and 9. Figure S9.1: lambda = 0.1. Figure S9.2: lambda = 0.4. Figure S9.3: mutRate = 0.0001. Figure S9.4: mutRate = 0.01. Figure S9.5: rndKill = 0.01. Figure S9.6: rndKill = 0.1. Figure S9.7: daughtersAlwaysLeave, daughtersAlwaysStay and numEnv = 5. Lowering the rate to switch from recovery phase to stress phase to 0.1 led to the evolution of a genotype with a high basal protection as was observed with a lambda of 0.2 (compare Figure S9.1a to Fig. 7a). But we did no longer observe the evolution of a memory genotype (compare Figure S9.1b to Fig. 7b and Figure S9.1c to Fig. 9a). Interestingly a phenotype that segregated cellular protection only to one of the two cells emerging from division still did evolve in the case of two environments (Figure S9.1d). The simulation trajectories were comparable to the reference when lambda was increased from 0.2 of 0.4 (compare Figs. 7 and 9 to Figure S9.2). Qualitatively we observed the same simulation outcome when decreasing the mutation rate from 0.01 to 0.0001, although a slower convergence was observed (compare Figs. 7 and 9 to Figure S9.3). Simulation results that were run with an increased mutation rate (0.1) diverged from what we observed in the reference simulations (compare Figs. 7 and 9 to [file 12862_2017_884_MOESM5_ESM.zip › FigureS9_6.png]

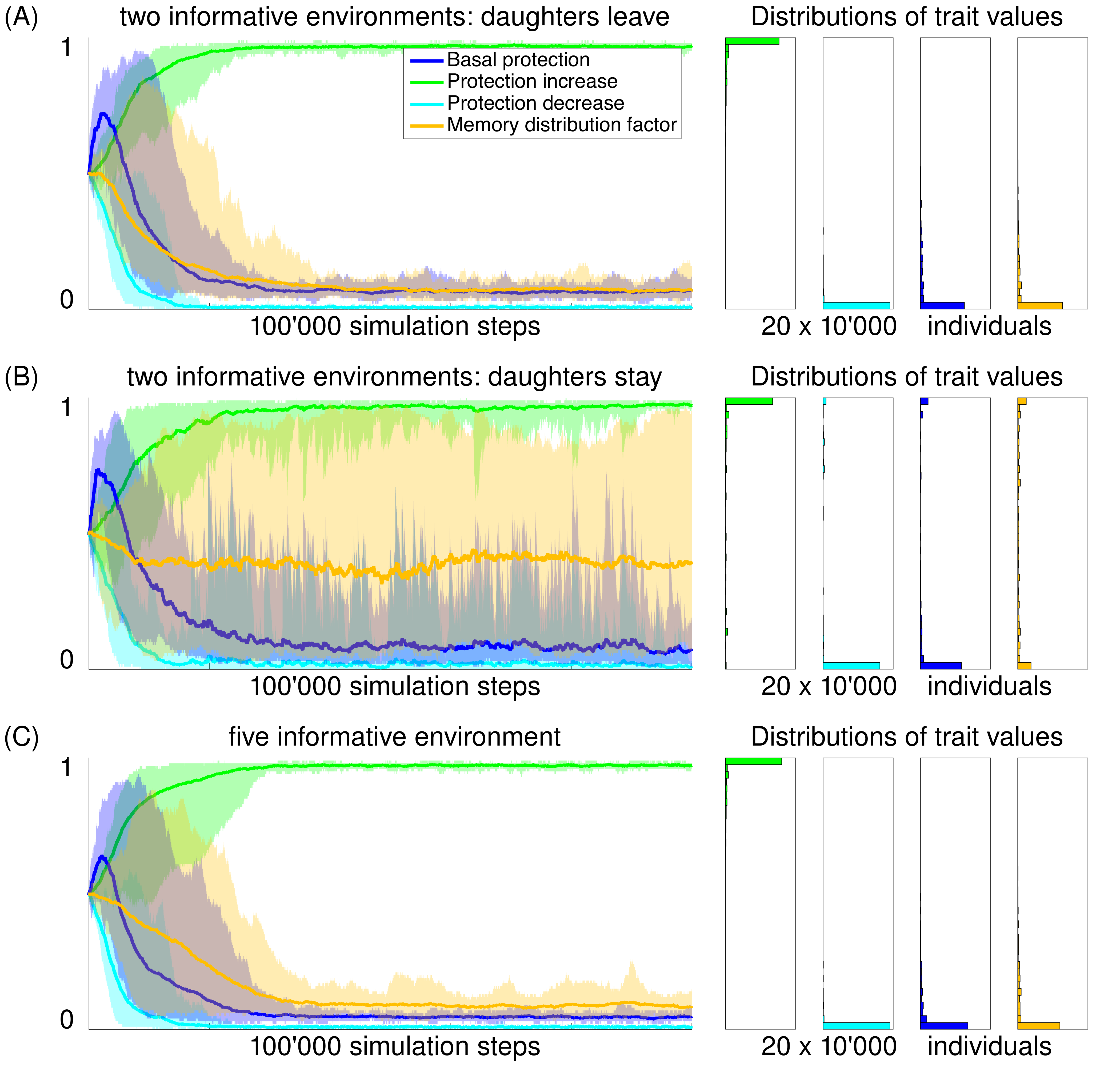

Supplement: Supplementary file 5 — To assess the sensitivity of the simulation outcomes to varying simulation parameters, we changed a single simulation parameter at a time and rerun the simulations shown in Fig. 7 and 9. Since we did not vary all of the parameters and did not change more than one parameter at a time, this is not exhaustive. The following table lists the parameter values used in the simulations in Fig. 5 and 7 in the column Default. For each parameter we chose a lower and a higher value to rerun the simulation (columns Lower and Higher). See description and usage of the parameters in supplementary material S7. The following figures show the results from simulations where single parameters were changed compared to the reference parameters used in Figs. 7 and 9. Figure S9.1: lambda = 0.1. Figure S9.2: lambda = 0.4. Figure S9.3: mutRate = 0.0001. Figure S9.4: mutRate = 0.01. Figure S9.5: rndKill = 0.01. Figure S9.6: rndKill = 0.1. Figure S9.7: daughtersAlwaysLeave, daughtersAlwaysStay and numEnv = 5. Lowering the rate to switch from recovery phase to stress phase to 0.1 led to the evolution of a genotype with a high basal protection as was observed with a lambda of 0.2 (compare Figure S9.1a to Fig. 7a). But we did no longer observe the evolution of a memory genotype (compare Figure S9.1b to Fig. 7b and Figure S9.1c to Fig. 9a). Interestingly a phenotype that segregated cellular protection only to one of the two cells emerging from division still did evolve in the case of two environments (Figure S9.1d). The simulation trajectories were comparable to the reference when lambda was increased from 0.2 of 0.4 (compare Figs. 7 and 9 to Figure S9.2). Qualitatively we observed the same simulation outcome when decreasing the mutation rate from 0.01 to 0.0001, although a slower convergence was observed (compare Figs. 7 and 9 to Figure S9.3). Simulation results that were run with an increased mutation rate (0.1) diverged from what we observed in the reference simulations (compare Figs. 7 and 9 to [file 12862_2017_884_MOESM5_ESM.zip › FigureS9_7.png]

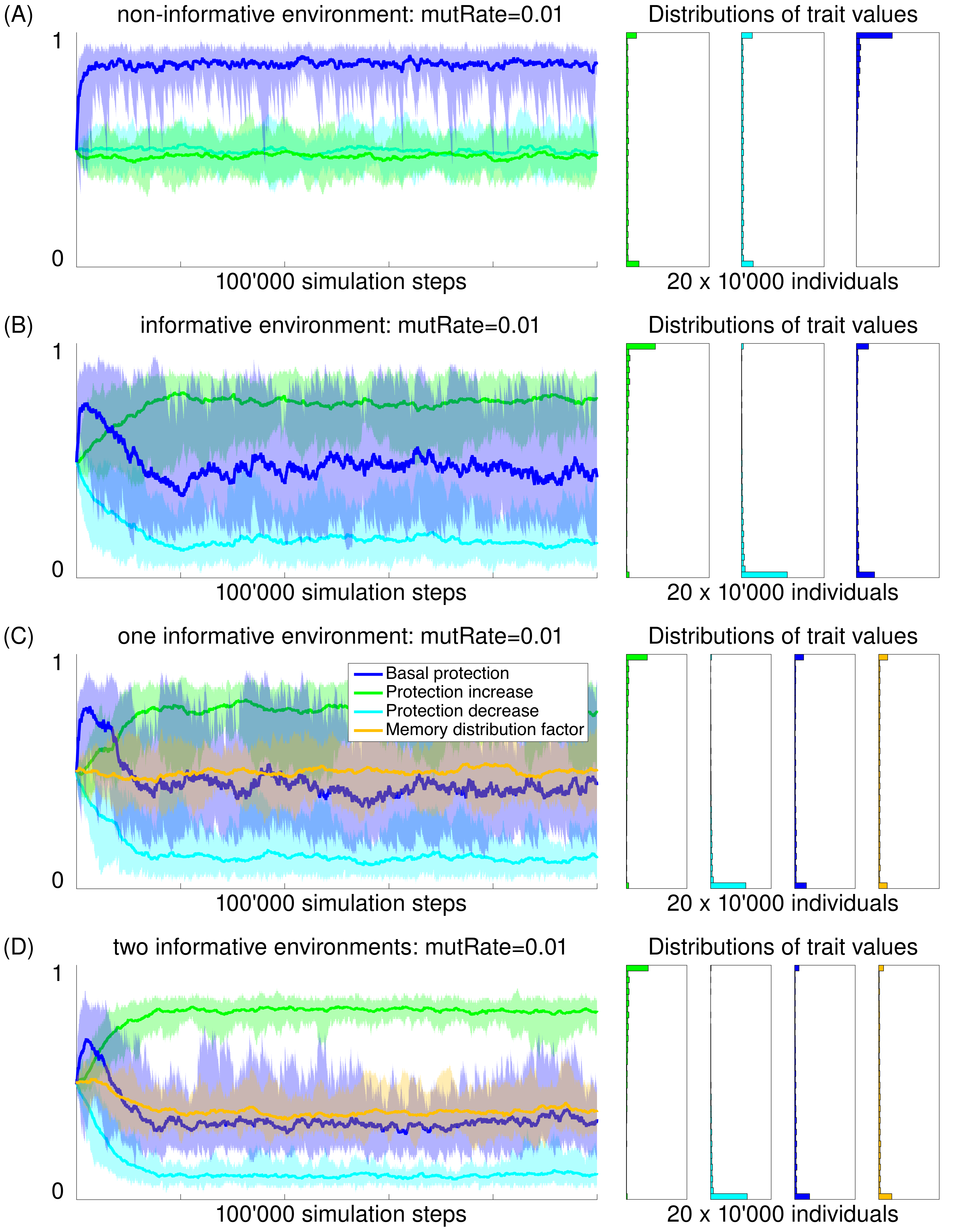

Supplement: Supplementary file 5 — To assess the sensitivity of the simulation outcomes to varying simulation parameters, we changed a single simulation parameter at a time and rerun the simulations shown in Fig. 7 and 9. Since we did not vary all of the parameters and did not change more than one parameter at a time, this is not exhaustive. The following table lists the parameter values used in the simulations in Fig. 5 and 7 in the column Default. For each parameter we chose a lower and a higher value to rerun the simulation (columns Lower and Higher). See description and usage of the parameters in supplementary material S7. The following figures show the results from simulations where single parameters were changed compared to the reference parameters used in Figs. 7 and 9. Figure S9.1: lambda = 0.1. Figure S9.2: lambda = 0.4. Figure S9.3: mutRate = 0.0001. Figure S9.4: mutRate = 0.01. Figure S9.5: rndKill = 0.01. Figure S9.6: rndKill = 0.1. Figure S9.7: daughtersAlwaysLeave, daughtersAlwaysStay and numEnv = 5. Lowering the rate to switch from recovery phase to stress phase to 0.1 led to the evolution of a genotype with a high basal protection as was observed with a lambda of 0.2 (compare Figure S9.1a to Fig. 7a). But we did no longer observe the evolution of a memory genotype (compare Figure S9.1b to Fig. 7b and Figure S9.1c to Fig. 9a). Interestingly a phenotype that segregated cellular protection only to one of the two cells emerging from division still did evolve in the case of two environments (Figure S9.1d). The simulation trajectories were comparable to the reference when lambda was increased from 0.2 of 0.4 (compare Figs. 7 and 9 to Figure S9.2). Qualitatively we observed the same simulation outcome when decreasing the mutation rate from 0.01 to 0.0001, although a slower convergence was observed (compare Figs. 7 and 9 to Figure S9.3). Simulation results that were run with an increased mutation rate (0.1) diverged from what we observed in the reference simulations (compare Figs. 7 and 9 to [file 12862_2017_884_MOESM5_ESM.zip › FigureS9_4.png]

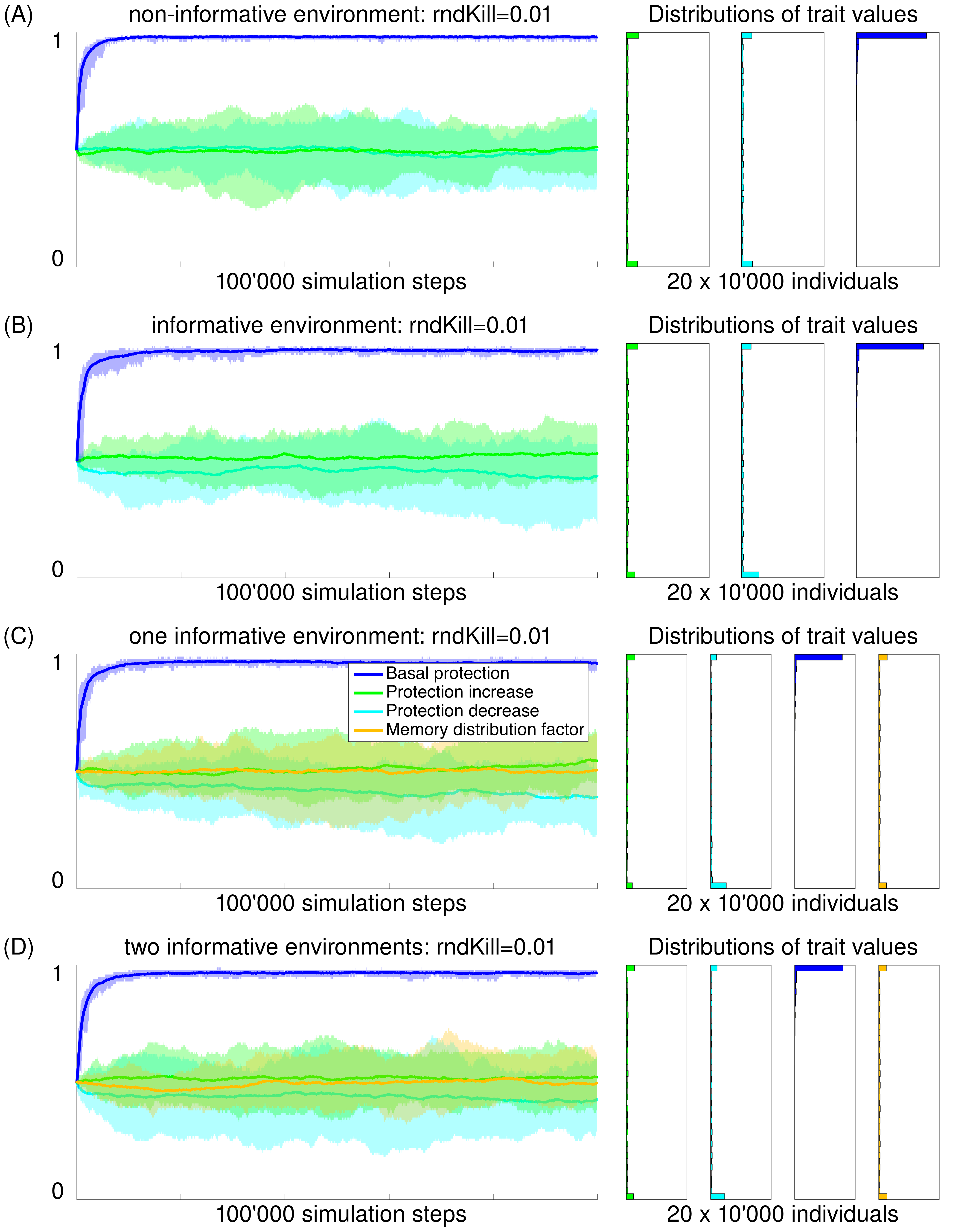

Supplement: Supplementary file 5 — To assess the sensitivity of the simulation outcomes to varying simulation parameters, we changed a single simulation parameter at a time and rerun the simulations shown in Fig. 7 and 9. Since we did not vary all of the parameters and did not change more than one parameter at a time, this is not exhaustive. The following table lists the parameter values used in the simulations in Fig. 5 and 7 in the column Default. For each parameter we chose a lower and a higher value to rerun the simulation (columns Lower and Higher). See description and usage of the parameters in supplementary material S7. The following figures show the results from simulations where single parameters were changed compared to the reference parameters used in Figs. 7 and 9. Figure S9.1: lambda = 0.1. Figure S9.2: lambda = 0.4. Figure S9.3: mutRate = 0.0001. Figure S9.4: mutRate = 0.01. Figure S9.5: rndKill = 0.01. Figure S9.6: rndKill = 0.1. Figure S9.7: daughtersAlwaysLeave, daughtersAlwaysStay and numEnv = 5. Lowering the rate to switch from recovery phase to stress phase to 0.1 led to the evolution of a genotype with a high basal protection as was observed with a lambda of 0.2 (compare Figure S9.1a to Fig. 7a). But we did no longer observe the evolution of a memory genotype (compare Figure S9.1b to Fig. 7b and Figure S9.1c to Fig. 9a). Interestingly a phenotype that segregated cellular protection only to one of the two cells emerging from division still did evolve in the case of two environments (Figure S9.1d). The simulation trajectories were comparable to the reference when lambda was increased from 0.2 of 0.4 (compare Figs. 7 and 9 to Figure S9.2). Qualitatively we observed the same simulation outcome when decreasing the mutation rate from 0.01 to 0.0001, although a slower convergence was observed (compare Figs. 7 and 9 to Figure S9.3). Simulation results that were run with an increased mutation rate (0.1) diverged from what we observed in the reference simulations (compare Figs. 7 and 9 to [file 12862_2017_884_MOESM5_ESM.zip › FigureS9_5.png]

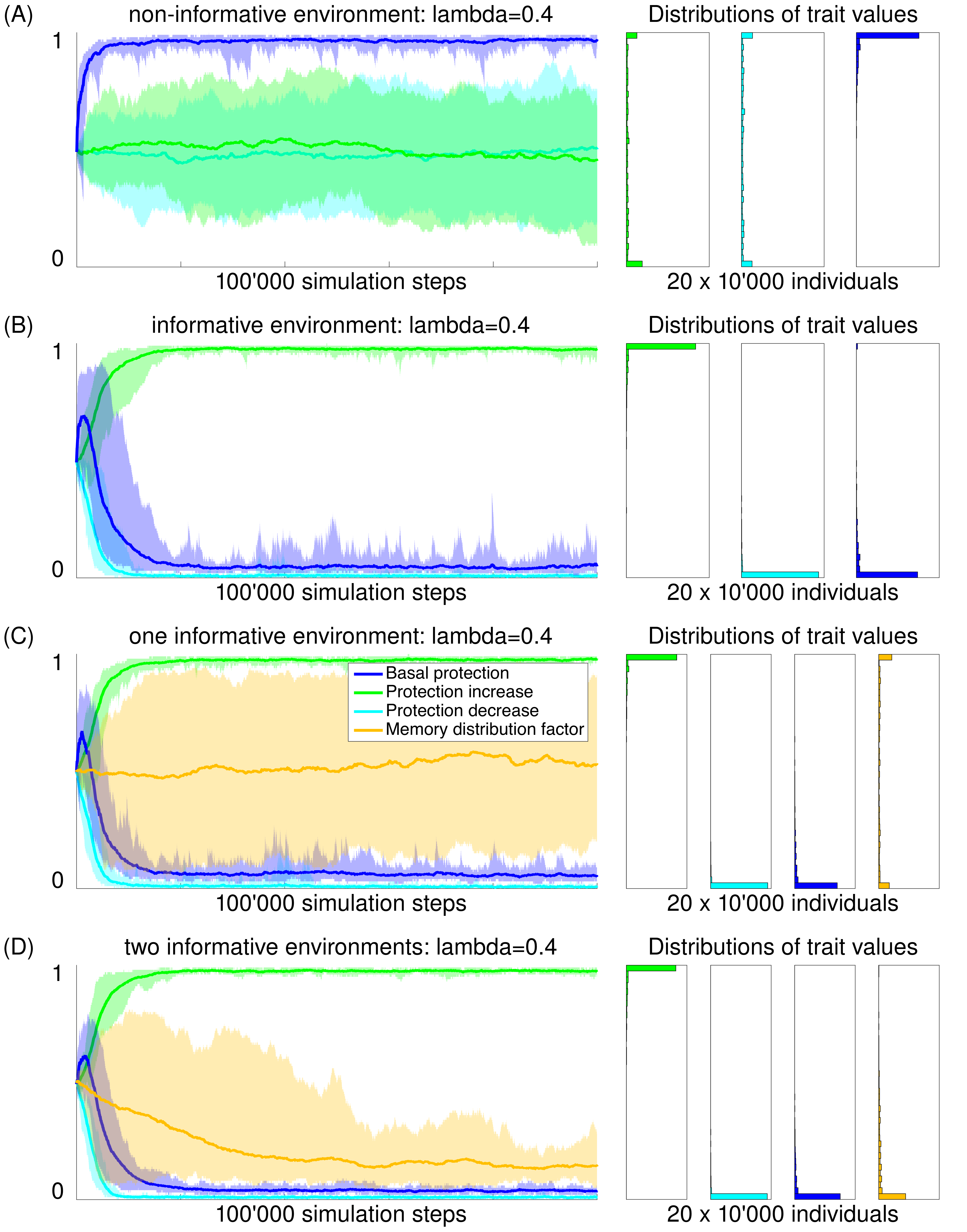

Supplement: Supplementary file 5 — To assess the sensitivity of the simulation outcomes to varying simulation parameters, we changed a single simulation parameter at a time and rerun the simulations shown in Fig. 7 and 9. Since we did not vary all of the parameters and did not change more than one parameter at a time, this is not exhaustive. The following table lists the parameter values used in the simulations in Fig. 5 and 7 in the column Default. For each parameter we chose a lower and a higher value to rerun the simulation (columns Lower and Higher). See description and usage of the parameters in supplementary material S7. The following figures show the results from simulations where single parameters were changed compared to the reference parameters used in Figs. 7 and 9. Figure S9.1: lambda = 0.1. Figure S9.2: lambda = 0.4. Figure S9.3: mutRate = 0.0001. Figure S9.4: mutRate = 0.01. Figure S9.5: rndKill = 0.01. Figure S9.6: rndKill = 0.1. Figure S9.7: daughtersAlwaysLeave, daughtersAlwaysStay and numEnv = 5. Lowering the rate to switch from recovery phase to stress phase to 0.1 led to the evolution of a genotype with a high basal protection as was observed with a lambda of 0.2 (compare Figure S9.1a to Fig. 7a). But we did no longer observe the evolution of a memory genotype (compare Figure S9.1b to Fig. 7b and Figure S9.1c to Fig. 9a). Interestingly a phenotype that segregated cellular protection only to one of the two cells emerging from division still did evolve in the case of two environments (Figure S9.1d). The simulation trajectories were comparable to the reference when lambda was increased from 0.2 of 0.4 (compare Figs. 7 and 9 to Figure S9.2). Qualitatively we observed the same simulation outcome when decreasing the mutation rate from 0.01 to 0.0001, although a slower convergence was observed (compare Figs. 7 and 9 to Figure S9.3). Simulation results that were run with an increased mutation rate (0.1) diverged from what we observed in the reference simulations (compare Figs. 7 and 9 to [file 12862_2017_884_MOESM5_ESM.zip › FigureS9_2.png]

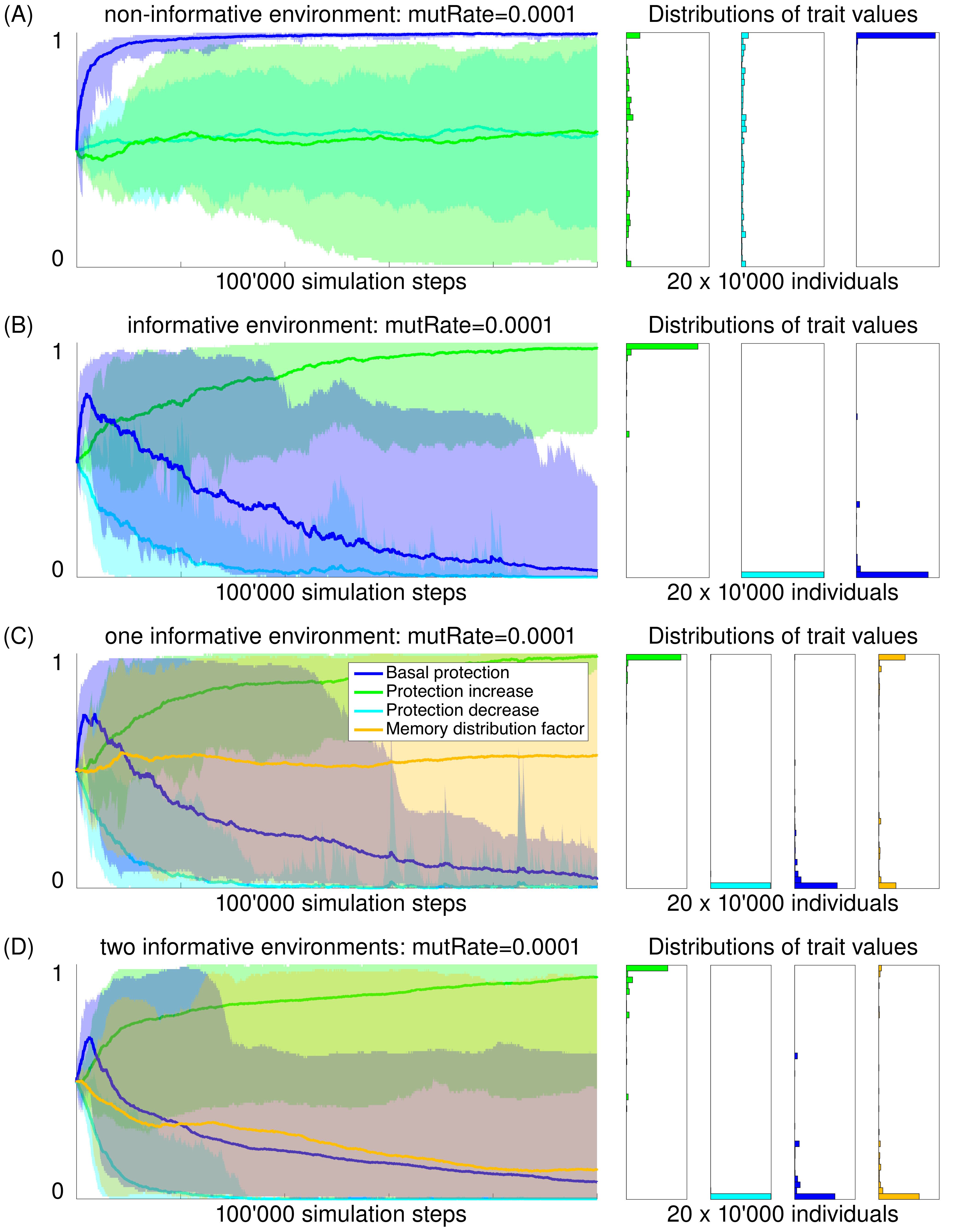

Supplement: Supplementary file 5 — To assess the sensitivity of the simulation outcomes to varying simulation parameters, we changed a single simulation parameter at a time and rerun the simulations shown in Fig. 7 and 9. Since we did not vary all of the parameters and did not change more than one parameter at a time, this is not exhaustive. The following table lists the parameter values used in the simulations in Fig. 5 and 7 in the column Default. For each parameter we chose a lower and a higher value to rerun the simulation (columns Lower and Higher). See description and usage of the parameters in supplementary material S7. The following figures show the results from simulations where single parameters were changed compared to the reference parameters used in Figs. 7 and 9. Figure S9.1: lambda = 0.1. Figure S9.2: lambda = 0.4. Figure S9.3: mutRate = 0.0001. Figure S9.4: mutRate = 0.01. Figure S9.5: rndKill = 0.01. Figure S9.6: rndKill = 0.1. Figure S9.7: daughtersAlwaysLeave, daughtersAlwaysStay and numEnv = 5. Lowering the rate to switch from recovery phase to stress phase to 0.1 led to the evolution of a genotype with a high basal protection as was observed with a lambda of 0.2 (compare Figure S9.1a to Fig. 7a). But we did no longer observe the evolution of a memory genotype (compare Figure S9.1b to Fig. 7b and Figure S9.1c to Fig. 9a). Interestingly a phenotype that segregated cellular protection only to one of the two cells emerging from division still did evolve in the case of two environments (Figure S9.1d). The simulation trajectories were comparable to the reference when lambda was increased from 0.2 of 0.4 (compare Figs. 7 and 9 to Figure S9.2). Qualitatively we observed the same simulation outcome when decreasing the mutation rate from 0.01 to 0.0001, although a slower convergence was observed (compare Figs. 7 and 9 to Figure S9.3). Simulation results that were run with an increased mutation rate (0.1) diverged from what we observed in the reference simulations (compare Figs. 7 and 9 to [file 12862_2017_884_MOESM5_ESM.zip › FigureS9_3.png]

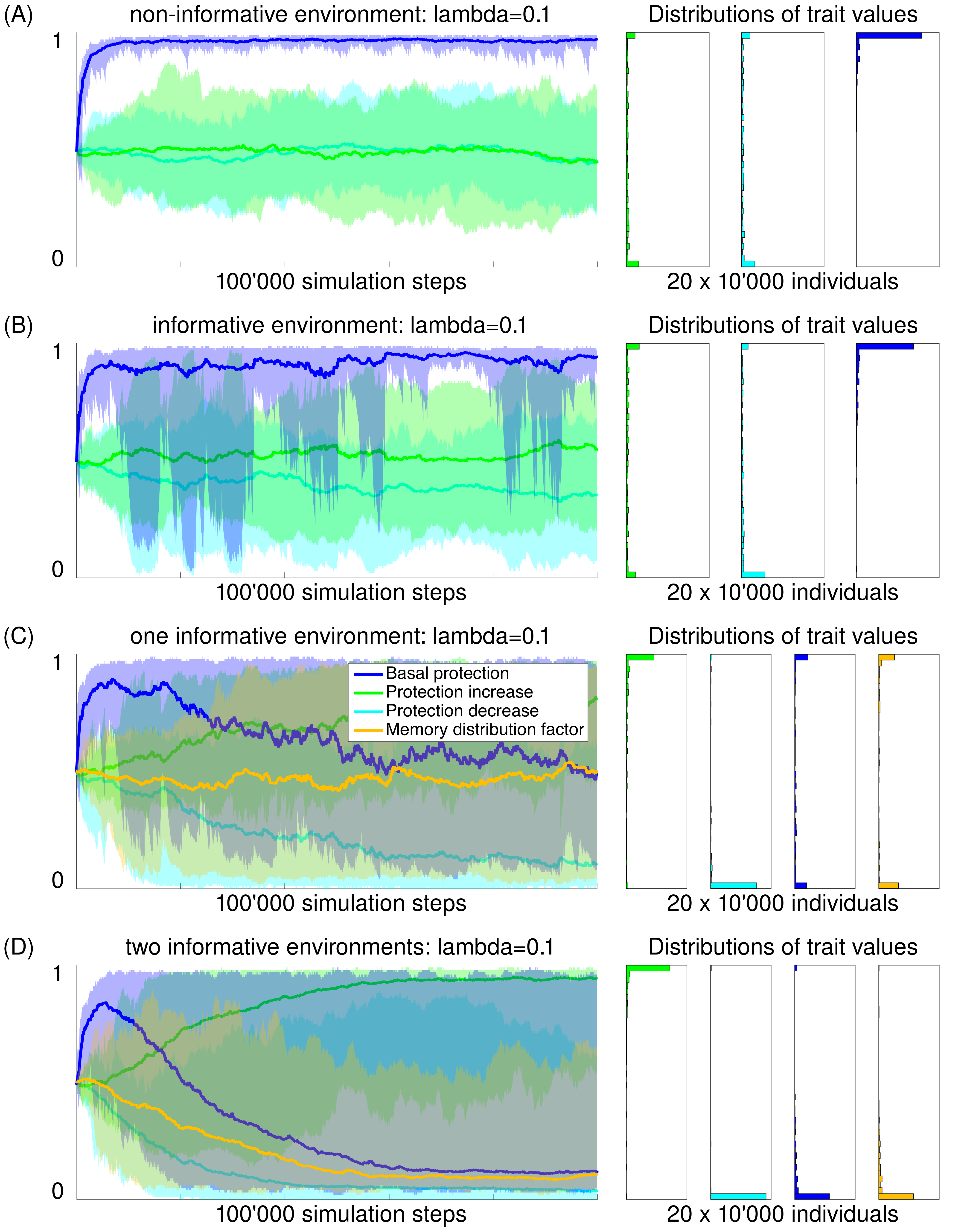

Supplement: Supplementary file 5 — To assess the sensitivity of the simulation outcomes to varying simulation parameters, we changed a single simulation parameter at a time and rerun the simulations shown in Fig. 7 and 9. Since we did not vary all of the parameters and did not change more than one parameter at a time, this is not exhaustive. The following table lists the parameter values used in the simulations in Fig. 5 and 7 in the column Default. For each parameter we chose a lower and a higher value to rerun the simulation (columns Lower and Higher). See description and usage of the parameters in supplementary material S7. The following figures show the results from simulations where single parameters were changed compared to the reference parameters used in Figs. 7 and 9. Figure S9.1: lambda = 0.1. Figure S9.2: lambda = 0.4. Figure S9.3: mutRate = 0.0001. Figure S9.4: mutRate = 0.01. Figure S9.5: rndKill = 0.01. Figure S9.6: rndKill = 0.1. Figure S9.7: daughtersAlwaysLeave, daughtersAlwaysStay and numEnv = 5. Lowering the rate to switch from recovery phase to stress phase to 0.1 led to the evolution of a genotype with a high basal protection as was observed with a lambda of 0.2 (compare Figure S9.1a to Fig. 7a). But we did no longer observe the evolution of a memory genotype (compare Figure S9.1b to Fig. 7b and Figure S9.1c to Fig. 9a). Interestingly a phenotype that segregated cellular protection only to one of the two cells emerging from division still did evolve in the case of two environments (Figure S9.1d). The simulation trajectories were comparable to the reference when lambda was increased from 0.2 of 0.4 (compare Figs. 7 and 9 to Figure S9.2). Qualitatively we observed the same simulation outcome when decreasing the mutation rate from 0.01 to 0.0001, although a slower convergence was observed (compare Figs. 7 and 9 to Figure S9.3). Simulation results that were run with an increased mutation rate (0.1) diverged from what we observed in the reference simulations (compare Figs. 7 and 9 to [file 12862_2017_884_MOESM5_ESM.zip › FigureS9_1.png]

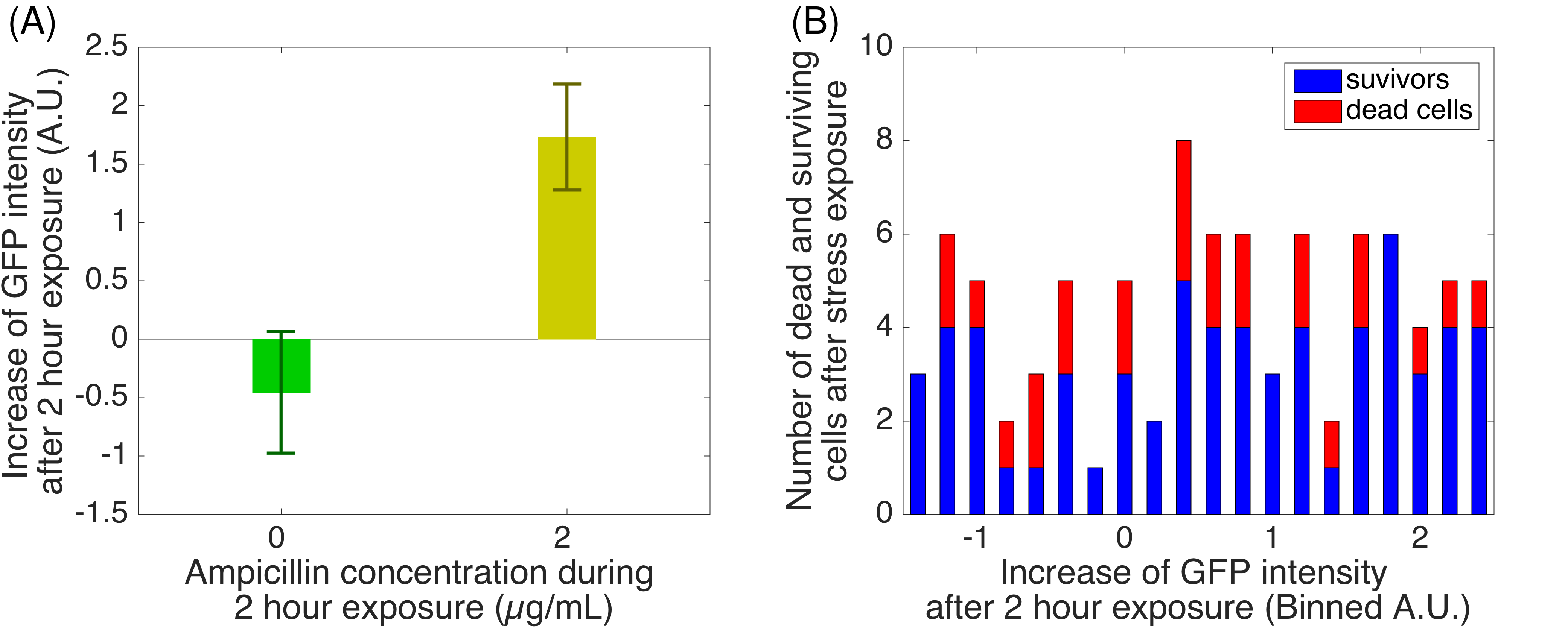

Supplement: Supplementary file 6 — It has been reported that the blaA (CC2139) gene is a major contributor to ampicillin resistance in C. crescentus [22]. We measured expression of blaA using a transcriptional reporter (see Methods). GFP (green fluorescent protein) intensity, a proxy for transcriptional activity of blaA, was measured before (t1) and after (t2) the warning event (0 or 10 µg/mL ampicillin for 2 h). After background correction the intensity level at t2 was subtracted from the intensity level at t1. (A) The mean of the differences is represented by the bars (error bars denote standard errors of the mean). For both conditions the intensity levels at t1 was compared to the intensity levels at t2 using a paired t-test statistic (N = 117 and 119 for 0 and 10 µg/mL ampicillin exposure). When cells were exposed to 10 µg/mL ampicillin for 2 h (yellow), the increase of the measured GFP intensity was significant (p < 0.01), while for the unwarned cells (green) no significant changes were observed (p = 0.3). (B) Numbers of surviving and dead cells are reported for small intervals of GFP intensity values (20 bins from -1.5 to 2.5 with size 0.2). No statistically significant association of GFP levels with survival after stress (logistic regression p = 0.68) could be established. (PNG 272 kb) [file 12862_2017_884_MOESM6_ESM.png]
