# Supplementary material for: Asymmetric cellular memory in bacteria exposed to antibiotics
Source: BMC Evol Biol. 2017 Mar 9;17:73. doi: 10.1186/s12862-017-0884-4 (PMC5343395; doi:10.1186/s12862-017-0884-4)
Supplement: Supplementary file 5 — To assess the sensitivity of the simulation outcomes to varying simulation parameters, we changed a single simulation parameter at a time and rerun the simulations shown in Fig. 7 and 9. Since we did not vary all of the parameters and did not change more than one parameter at a time, this is not exhaustive. The following table lists the parameter values used in the simulations in Fig. 5 and 7 in the column Default. For each parameter we chose a lower and a higher value to rerun the simulation (columns Lower and Higher). See description and usage of the parameters in supplementary material S7. The following figures show the results from simulations where single parameters were changed compared to the reference parameters used in Figs. 7 and 9. Figure S9.1: lambda = 0.1. Figure S9.2: lambda = 0.4. Figure S9.3: mutRate = 0.0001. Figure S9.4: mutRate = 0.01. Figure S9.5: rndKill = 0.01. Figure S9.6: rndKill = 0.1. Figure S9.7: daughtersAlwaysLeave, daughtersAlwaysStay and numEnv = 5. Lowering the rate to switch from recovery phase to stress phase to 0.1 led to the evolution of a genotype with a high basal protection as was observed with a lambda of 0.2 (compare Figure S9.1a to Fig. 7a). But we did no longer observe the evolution of a memory genotype (compare Figure S9.1b to Fig. 7b and Figure S9.1c to Fig. 9a). Interestingly a phenotype that segregated cellular protection only to one of the two cells emerging from division still did evolve in the case of two environments (Figure S9.1d). The simulation trajectories were comparable to the reference when lambda was increased from 0.2 of 0.4 (compare Figs. 7 and 9 to Figure S9.2). Qualitatively we observed the same simulation outcome when decreasing the mutation rate from 0.01 to 0.0001, although a slower convergence was observed (compare Figs. 7 and 9 to Figure S9.3). Simulation results that were run with an increased mutation rate (0.1) diverged from what we observed in the reference simulations (compare Figs. 7 and 9 to [file 12862_2017_884_MOESM5_ESM.zip › Table S9.pdf]

S9: Sensitivity of the simulation outcomes to parameter values

Parameter	Default	Lower	Higher
recoveryStressSwitchRate (lambda)	0.2	0.1	0.4
mutRate	0.001	0.0001	0.01
rndKill	0.005	0.001	0.01
numEnv	2	-	5
daughtersAlwaysLeave	No	Yes	
daughtersAlwaysStay	No	Yes	
