# Supplementary material for: Asymmetric cellular memory in bacteria exposed to antibiotics
Source: BMC Evol Biol. 2017 Mar 9;17:73. doi: 10.1186/s12862-017-0884-4 (PMC5343395; doi:10.1186/s12862-017-0884-4)
Supplement: Supplementary file 7 — Additional tables and listings. (PDF 143 kb) [file 12862_2017_884_MOESM7_ESM.pdf]

Additional file 7

S4: Single-cell experiments: days, channels and conditions

The following table lists channels (of a microfluidic device) and experimental conditions that were applied for each experiment day.

Day	Channel ID	Condition	Number of cells
Day 1	1	No warning / Stress	152
	2	No warning / Stress	155
	5	Warning / Stress	166
	6	Warning / Stress	158
Day 2	1	No warning / Stress	156
	2	No warning / Stress	165
	5	Warning / Stress	151
	6	Warning / Stress	163
Day 3	3	No warning / Stress	144
	4	No warning / Stress	147
	7	Warning / Stress	133
	8	Warning / Stress	105
Day 4	2	Warning / Stress	136
	4	Warning / Stress	154
	6	No warning / Stress	137
	7	No warning / Stress	147

Warning: 10 μg / mL Ampicillin

Stress: 2000 μg / mL Ampicillin

S5: Linear model

We defined the following linear model to quantify survival dependent on cell type, warning intensity, cell cycle position and experiment day.

```
model <-  glm(survival ~  warnC*cellType + cyclePosType:poly(cyclePos,2) + expDay ,
data=data, family="binomial")
```

A binomial distribution was used to model the response variable survival. The effects of warning intensity and cell type were modeled as single effects and their interaction. Cell cycle state was modeled as an interaction between cyclePosType (2=cell about to divide for the first time versus 1=cell had divided previously) and cyclePos (corresponding to the time in units of 5 minutes since last division in the interval 5-70 minutes for cells that had divided previously, and the time since birth in the interval 5-90 minutes for cells that were about to divide for the first time). See supplementary material S3 for a description of the cell cycle state model. The experiment day was included to test for batch effects.

```
Coefficients:
                                Estimate Std. Error z value Pr(>|z|)
(Intercept)                      1.16954    0.12101   9.665  < 2e-16 ***
warnC10                          0.47320    0.12796   3.698 0.000217 ***
cellType2                       -0.01464    0.13836  -0.106 0.915712
expDay505                       -0.19947    0.13223  -1.509 0.131407
expDay519                       -0.13913    0.13956  -0.997 0.318775
expDay528                       -0.12564    0.13642  -0.921 0.357078
warnC10:cellType2               -0.39631    0.19575  -2.025 0.042916 *
cyclePosType1:poly(cyclePos, 2)1   4.46282    3.64984   1.223 0.221427
cyclePosType2:poly(cyclePos, 2)1 -27.53499    4.75616  -5.789 7.07e-09 ***
cyclePosType1:poly(cyclePos, 2)2  21.61555    4.06577   5.316 1.06e-07 ***
cyclePosType2:poly(cyclePos, 2)2  11.70189    3.79012   3.087 0.002019 **
---
Signif. codes:  0 '***' 0.001 '**' 0.01 '*' 0.05 '.' 0.1 ' ' 1
```

Not only affected the warning intensity survival, but cell type interacted with this effect. The experiment day had no significant effect on survival.

Of note, we found no evidence for a statistical association in survival between mothers and daughters: daughters whose mother survived the stress event were not significantly more (or less) likely to also survive than daughters whose mother died (Chi-square tests of independence were

performed to examine the relation between unwarned mothers and their corresponding daughters, N=433, X^2=0.013, p=0.91 and between warned mothers and their corresponding daughters N=438, X^2=2.66, p=0.10).

S7: Parameters and pseudocode of the evolution simulation

The following table lists the parameters that were used in the model.

Simulation Parameters

Name	Default or bounds	Type	Description
currentP	[0, 1]	Phenotype	Current protection level of a cell
basalProtection	[0, 1]	Genotypic trait	Minimal protection level to maintain
protectionDecrease	[0, 1]	Genotypic trait	The amount of protection to subtract from the currentP at each time step
protectionIncrease	[0, 1]	Genotypic trait	The amount of protection to add to the currentP at each time step
memoryDistributionFactor	[0, 1]	Genotypic trait	The fraction of currentP the daughter cell inherits from the mother cell upon division. For the simulations described in Figure 6 and 7 the memoryDistributionFactor was fixed to 0.5. For the simulations described in Figure 8 and 9, it was free to evolve.
numEnv	1 or 2	Parameter	The number of environments modeled. numEnv was 1 for simulations described in Figures 6, 7, 8A, 9A and numEnv was 2 for simulations described in Figure 8B and 9B. NumEnv was set to 5 in the supplementary Figure S9.7C.
numTimeSteps	100'000	Parameter	Number of time steps each simulation was run.
popSize	10'000	Parameter	10’000 individuals were initialized at the start of each simulation. popSize also marks the carrying capacity during the simulation run.
killRate	0.05	Parameter	Fraction of the population killed in

			each round.
mutRate	0.001	Parameter	Fraction of the population chosen to mutate in each round by drawing from a standard distribution with the mean being the current trait value and a standard deviation of 0.2.
randomWarnSwitchRate	0.005	Parameter	Rate to switch from favorable condition to warning condition (Figure 6D and 6F).
randomStressSwitchRate	0.004	Parameter	Rate to switch from favorable to stress conditions (Figure 6D).
warnRecoverySwitchRate	0.8	Parameter	Rate to switch from warning to post-warning favorable condition (only in the informative model, see Figure 6E).
recoveryStressSwitchRate (lambda)	0.2	Parameter	Rate to switch from the recovery phase (post-warning favorable condition) to stress condition (only in informative model, see Figure 6E). In the text we refer to this parameter as 'lambda'.
daughtersAlwaysStay / daughtersAlwaysLeave	No / No	Parameters	This is only relevant in simulations with numEnv > 1: The default behavior is that daughter cells are assigned to one of the environments with equal probability. If daughtersAlwaysStay is Yes, the daughters will stay in the same environment. If daughtersAlwaysLeave is Yes, the daughters will always be moved to a different environment.

Simulation steps

The following pseudocode illustrates the order of instructions that was followed when running the in silico evolution experiments (see Figure 5A for a graphical representation).

- Initialize
  - Generate numEnv environments of type informative or non-informative
    - Generate vector of length numTimeSteps using the model described in Figure 6D and E. Set environmental condition to 0, 1 or 2 indicating favorable, warning and stress conditions.

- Generate popSize individuals for each environment. For each individual:
    - Initialize currentP: Randomly choose a value between 0 and 1
    - For each trait (basalProtection, protectionDecrease, protectionIncrease [and memoryDistributionFactor when we state that it is evolvable, otherwise it is set to 0.5])
        - Initialize trait: Randomly choose a value between 0 and 1
- Simulate numTimeSteps. For each time step:
    - Check survival:
        - In all environments: survive if randomNumber > 0.05
        - If in a stress environment: Survive if randomNumber > currentP
    - Adjust protection
        - currentP = currentP - protectionDecrease
        - currentP = 0, if currentP < 0
        - currentP = basalProtection, if currentP < basalProtection
        - currentP = currentP + protectionIncrease
        - currentP = 1, if currentP > 1
    - Select reproducers
        - Add individual to group of potential reproducers if randomNumber < currentP
        - Draw randomly reproducers from the group of potential reproducers until the carrying capacity is reached (10'000 individuals per environment)
    - Reproduce
        - For all members of the group of reproducers:
            - Copy traits of reproducing cell (mother cell) to daughter cell
            - Split currentP according to memoryDistributionFactor

            - currentP_motherCell = memoryDistributionFactor * currentP
            - currentP_daughterCell = currentP - currentP_motherCell
- Mutate
    - Select cell for mutation if randomNumber < mutRate
        - Select one of the traits randomly
            - Draw a new value for this trait by drawing from a normal distribution with current trait value as the mean and a standard deviation of 0.2
            - 0, if new value < 0
            - 1, if new value > 1
